# Determination of Configuration and Conformation of a Reserpine Derivative with Seven Stereogenic Centers Using Molecular Dynamics with RDC‐Derived Tensorial Constraints**

**DOI:** 10.1002/chem.202002642

**Published:** 2020-10-01

**Authors:** Emine Sager, Pavleta Tzvetkova, Alvar D. Gossert, Philippe Piechon, Burkhard Luy

**Affiliations:** ^1^ Institut für Organische Chemie Karlsruher Institut für Technologie (KIT) Fritz-Haber-Weg 6 76131 Karlsruhe Germany; ^2^ Novartis Pharma AG Novartis Institutes for Biomedical Research 4002 Basel Switzerland; ^3^ Institut für Biologische Grenzflächen 4—Magnetische Resonanz Karlsruher Institut für Technologie (KIT) Postfach 3640 76021 Karlsruhe Germany; ^4^ Institut für Molekularbiologie und Biophysik ETH Zürich 8093 Zürich Switzerland

**Keywords:** molecular dynamics, natural products, quinolizidine, reserpine, residual dipolar couplings, structure elucidation

## Abstract

NMR‐based determination of the configuration of complex molecules containing many stereocenters is often not possible using traditional NOE data and coupling patterns. Making use of residual dipolar couplings (RDCs), we were able to determine the relative configuration of a natural product containing seven stereocenters, including a chiral amine lacking direct RDC data. To identify the correct relative configuration out of 32 possible ones, experimental RDCs were used in three different approaches for data interpretation: by fitting experimental data based singular value decomposition (SVD) using a single alignment tensor and either (i) a single conformer or (ii) multiple conformers, or alternatively (iii) using molecular dynamics simulations with tensorial orientational constraints (MDOC). Even though in all three approaches one and the same configuration could be selected and clear discrimination between possible configurations was achieved, the experimental data was not fully satisfied by the methods based on single tensor approaches. While these two approaches are faster, only MDOC is able to fully reproduce experimental results, as the obtained conformational ensemble adequately covers the conformational space necessary to describe the molecule with inherent flexibility.

## Graphical abstract

**Use RDCs**! Residual dipolar couplings (RDCs) were used to solve the relative configuration of a reserpine derivative with seven stereogenic centers, including a tertiary amine. Three different methods were tested for data interpretation, including the MDOC (molecular dynamics simulations with tensorial orientational constraints) approach, which also provides a conformational ensemble of the flexible molecule that fulfils all experimental data. The configuration has been confirmed independently by X‐ray diffraction.

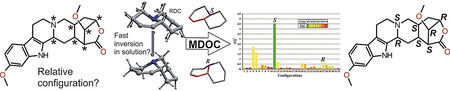

## Introduction

Natural products play a critical role in drug discovery, as they provide access to new chemotypes and structural diversity. The substances are extracted and isolated from different living organisms and tested for activity in assays against molecular targets of diseases. In order to take advantage of natural products as potential drug candidates, their exact chemical structure and configuration needs to be known. This knowledge will allow to re‐synthetize them in larger quantities and gain access to structural isomers. Structure and configuration determination can be achieved by X‐ray crystallography, if sufficient material is available and diffracting crystals are obtained. Unfortunately, this is a time‐consuming process and in many cases it is not possible at all. Therefore, NMR spectroscopy is the more prevalent technique for determining the chemical structure of natural products. However, investigating the configuration of molecules containing many stereocenters is often not possible based on traditional NOE data and coupling patterns. In such cases, complementary NMR data needs to be recorded and interpreted. In recent years, residual dipolar couplings have become an attractive source of such additional data,^[1]^ which will be further exploited in this manuscript.

The reserpine derivative RD‐1 represents a case, where the assignment of the relative stereochemistry was not possible based on traditional NMR analysis. Reserpine is a well‐known natural product: it is an alkaloid found in the roots of *Rauwolfia serpentina* and *Rauwolfia vomitoria* (for the structure of reserpine we refer the reader to the supporting information)^[2]^ Pseudoreserpic acid derivatives are useful as sedatives and antihypertensives.^[3]^ The core of these molecules contains a tertiary amine, which is part of a quinolizidine system, that is, two fused cyclohexane rings with a nitrogen at the bridgehead position.

**Figure 1:**
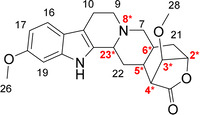
Reserpine derivate (RD‐1)—chemical structure and numbering used. Note that the numbering scheme deviates from the IUPAC numbering of the compound.

The investigated reserpine derivative (RD‐1) has the systematic name (1*S*,4*R*,5a*S*,14b*R*,15a*S*,16*R*)‐12,16‐dimethoxy‐4,5,5a,6,8,9,14,14b,15,15a‐decahydro‐1,4‐methanoindolo[2,3‐a]oxepino[4,5‐g]quinolizin‐2(1*H*)‐one. The structure and the numbering used in this study are depicted in Figure 1.

In total, the molecule contains seven stereocenters including the tertiary amine, which is treated here as an additional chiral center. It is known that inversion of the nitrogen pyramid in amines can readily take place via a change in *sp* ^3^ hybridization of the nitrogen atom by passing through a planar *sp* ^2^ state[^3b^, ^4^] This inversion process in most amines is too fast to be detectable by NMR. However, in some cases suitable substituents can sufficiently stabilize the two interchanging states and allow the separation of the two isomers. For this molecule it was initially not obvious which of the cases applies, fast inversion or presence of just one conformation.

The aim of the study was the reliable determination of the relative stereochemistry of the reserpine derivative RD‐1 and the establishment of a protocol that could be applied in the same way to other, similar molecules. To achieve this, we also compared different computational methods for RDC‐based configurational analysis using state‐of‐the‐art approaches like SVD fitting as implemented in the program MSpin^[5]^ and molecular dynamics (MD) simulations with orientational RDC constraints (MDOC) as implemented in COSMOS.^[6]^ The latter program allows an effective coverage of all possible conformations in an 80 ns long MD simulation that fulfils all experimental constraints averaged over the resulting structural and orientational ensemble as good as possible.^[7]^ MSpin, in contrast, only works on single or few pre‐selected fixed 3D conformations of the molecules under study. Several different approaches are implemented in MSpin to fit or predict RDC data, of which three were tested here, namely: *single* conformer single alignment tensor fits and *multiple* conformers single alignment tensor fits, which are both based on singular value decomposition (SVD)^[8]^ and additionally the TRAMITE prediction, which uses an approximation of RDCs from tensors of inertia.^[9]^ From the three possible MSpin approaches as the gold standard in RDC‐based structure determination of small molecules, the last had to be excluded at an early stage, as the implemented prediction did not lead to good agreements with single optimized conformers, indicating that more complex prediction methods like the one proposed by Frank et al.^[10]^ or by Ibánez de Opakua et al.^[11]^ might be advisable.

In this manuscript, we show that the determination of the relative stereochemistry of the seven stereocenters reserpine derivative RD‐1−a relatively complex molecule which exhibits a certain degree of flexibility—can be achieved by the applicable MSpin and COSMOS RDC analysis methods; however, with significant differences concerning the fulfilment of experimental constraints and the related treatment of the inherent flexibility in RD‐1. We address the potential inversion of the tertiary amine by ^1^ *J*_CH_ coupling constants, its stereochemistry by ^1^ *D*_CH_ couplings, and give a detailed comparison of the static versus the molecular dynamics‐based approaches as tools for the analysis of relative configuration and conformation of complex molecules with inherent flexibility. The configurational assignment is verified with X‐ray analysis which was performed independently.

## Results and Discussion

### Experimental RDC data

**Figure 2:**
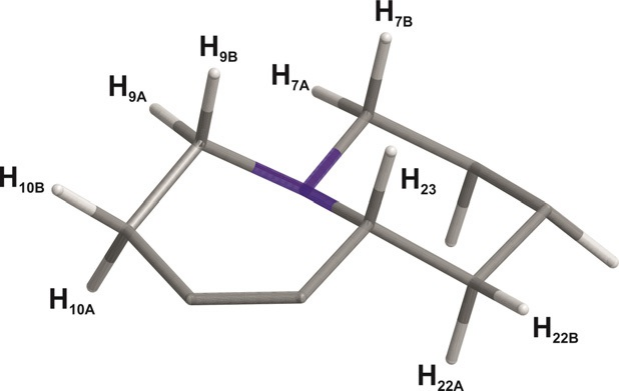
Central quinolizidine system of RD‐1, with numbering of relevant atoms.

After the assignment of all NMR‐active nuclei using standard procedures, residual dipolar coupling data were derived from the measured one‐bond coupling constants of the reserpine RD‐1 recorded under isotropic (^1^ *J*_CH_) and anisotropic (^1^ *T*_CH_=^1^ *J*_CH_+^1^ *D*_CH_) conditions, respectively. Partial alignment of the sample was achieved using a polymer‐based alignment medium. While a large number of polymer gels[^1c^, ^12^] and liquid crystalline phases^[13]^ have been reported in the literature, polymer gels in combination with a rubber‐based stretching device^[14]^ in our hands provide the best flexibility in adjusting the alignment strength. As [D_6_]DMSO was used as solvent for the isotropic measurements, we chose a polyacrylonitrile (PAN) gel[^12h^, ^12i^] as the alignment medium for the anisotropic sample. Anisotropic data were recorded at a deuterium quadrupolar splitting of Δν_Q_=6 Hz as a balanced compromise between signal width and residual dipolar coupling size. ^1^ *D*_CH_ couplings were calculated from the difference between the scalar couplings ^1^ *J*_CH_ of the isotropic sample and the total splitting of the anisotropic sample (^1^ *T*_CH_) as measured by CLIP‐HSQC^[15]^ and P.E.HSQC^[16]^ experiments. A total of 21 ^1^ *J*_CH_ and corresponding ^1^ *D*_CH_ couplings ranging from −25.6 to 32.1 Hz were thus accessible with errors ranging between 0.3 and 5 Hz as determined by the procedure for maximum error estimates as described by Kummerlöwe et al.^[17]^ (see Table S1). The experimental results for selected ^1^ *J*_CH_ couplings in CH and CH_2_ groups in proximity of the amine are also summarized in Figure 2 and Table 1. As both methoxy groups show averaged experimental values, corresponding RDCs were omitted in the single conformer fit and for better correspondence also in the multi conformer single alignment tensor fit.

**Table 1:** Experimental data for selected CH_2_ and CH groups around the amine.

Couplings	δ(^13^C) [ppm]^[a]^	δ(^1^H) [ppm]^[b]^	^1^ *J*_CH_ [Hz]^[c]^
C_9_H_9B_ (ax)	52.9	2.38	128.7
C_9_H_9A_ (eq)		2.93	136.4
C_7_H_7B_ (ax)	58.5	2.50	128.4
C_7_H_7A_ (eq)		2.62	137.1
C_23_H_23_	54.6	3.46	130.8
C_10_H_10B_ (ax)	21.8	2.53	127.0
C_10_H_10A_ (eq)		2.71	128.5
C_22_H_22B_ (eq)	35.0	2.35	127.8
C_22_H_22A_ (ax)		1.74	128.0

[a] Proton chemical shift in ppm; [b] Carbon chemical shift in ppm; [c] one bond CH scalar couplings in Hz.

### Treatment of the tertiary amine

The determination of the configuration at the amine N8 has not been possible with standard NMR methods in isotropic solution such as NOE‐derived distances and dihedral angles from ^3^ *J‐*couplings. The aliphatic CH_2_ and CH groups at positions 7, 9, and 23 provide a significant stabilization of the tertiary amine, but a potential rapid inversion cannot be excluded a priori. A theoretical structural search for conformations with inverted amines showed little differences in energies of the two isoforms and it was necessary to find a way to experimentally give evidence for the presence or absence of amine inversion.

The unambiguous solution to the problem was found as a side product of the one‐bond measurements: if rapid inversion would occur, all structural parameters like chemical shifts and especially scalar couplings of the neighboring aliphatic groups should be averaged. The experimentally determined ^1^ *J*_CH_ coupling constants of the axial protons of CH_2_ 7 and 9 (128.4 and 128.7 Hz, respectively) are much smaller than the ^1^ *J*_CH_ couplings of the corresponding equatorial protons (137.0 and 136.4 Hz, respectively).^[18]^ Equally, chemical shifts are significantly different for the protons within the CH_2_ 9, 2.93 ppm for the equatorial proton compared to 2.39 ppm for the axial proton. It can therefore be concluded that one configuration strongly dominates at the amine and potential inversion to a good approximation can be neglected.

### Generation of 32 possible diastereomers

RD‐1 contains 7 chiral centers. Two chiral centers (2 and 4 in Figure 1) are within a sterically hindered lactone ring, therefore only a reduced number of configurations is sterically accessible. In total, 32 relative configurations listed in Table 2 are possible, neglecting all enantiomers with *S‐*configuration at C2.

**Table 2:** Possible relative configurations of RD‐1.

Configurations^[a]^	C2C3C4C5C6N8C23^[b]^	Configurations^[a]^	C2C3C4C5C6N8C23^[b]^
1	*RRSSSSS*	17	*RRSSSRS*
2	*RSSSRSS*	18	*RSSSRRS*
3	*RRSSRSS*	19	*RRSSRRS*
4	*RRSRSSR*	20	*RRSRSRR*
5	*RSSRSSR*	21	*RSSRSRR*
6	*RSSSSSS*	22	*RSSSSRS*
7	*RRSRRSR*	23	*RRSRRRR*
8	*RSSRRSR*	24	*RSSRRRR*
9	*RSSRRSS*	25	*RSSRRRS*
10	*RRSRRSS*	26	*RRSRRRS*
11	*RRSSSSR*	27	*RRSSSRR*
12	*RSSSSSR*	28	*RSSSSRR*
13	*RSSRSSS*	29	*RSSRSRS*
14	*RRSRSSS*	30	*RRSRSRS*
15	*RRSSRSR*	31	*RRSSRRR*
16	*RSSSRSR*	32	*RSSSRRR*

[a] Running number identifying the configurations; [b] *R* and *S* identify the configuration of the stereocenters in the following sequence: C2 C3 C4 C5 C6 N8 C23. Note that for each relative configuration a mirror image (enantiomer) exists that is not listed and for which the same analysis can be applied.

Static structural models of the 32 different configurations have been generated with the program CORINA^[19]^ and subsequently optimized using the Schrödinger software package Release 2014‐2 (Maestro, Schrödinger, LLC, New York, NY, 2016). Corresponding 3D structures with minimal energies as well as ensembles of energy‐optimal structures were used for the following MSpin calculations and as initial structures for the COSMOS MDOC runs.

### Single conformer single alignment tensor SVD approach

Each geometry‐optimized configuration was fitted in MSpin to determine a global alignment tensor using the SVD option. Resulting RDCs were back‐calculated and compared with experimental data. The MSpin software package uses the Cornilescu Q^[20]^ as a fitting criterion. This quality factor allows the relative evaluation of best fitting structural models out of a predefined set. However, no absolute evaluation of the fulfillment of experimental constraints is possible. This can be much better achieved by the previously introduced quality factor (n/χ^2^),^[6c]^ which is explained further in the experimental section and has been used throughout the manuscript. A structural model that fully complies with experimental data within the experimental error must result in n/χ^2^>1; consequently, a value below 1 is a clear indication that at least one experimental value is outside its corresponding experimental error. In addition to the n/χ^2^ quality factor, we also give the number of such outliers for every structural model as another important number for evaluation. In this study we provide all experimental errors as maximum error estimates using the procedure defined by Kummerlöwe et al.^[17]^ As these error estimates correspond to a very high confidence level of approximately three times the standard deviation (3σ), a single outlier is in principle sufficient to falsify a structural model, as the model does not reproduce the experimental data within the error.

**Figure 3:**
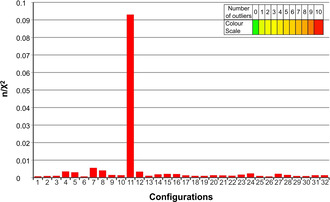
MSpin (SVD) quality factors n/χ^2^ calculated for all possible configurations of RD‐1 from a single conformer. On the horizontal axis, the 32 different configurations are listed using the numbering shown in Table 2. The color of the bar encodes the number of outliers that do not fulfil measured RDC values, as shown in the color scale on the upper right. Even the best static structural model has 10 RDCs that do not comply with the data within experimental errors.

**Figure 4:**
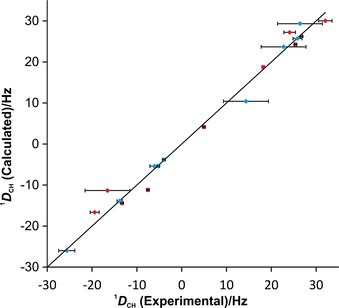
Plot of back‐calculated RDCs obtained with MSpin (SVD), against experimental RDCs for the best configuration 11 (*RRSSSSR*). Although the overall fit of experimental versus calculated data shows at first sight a good correlation, 10 out of the 19 RDCs are not reproduced within the error range of the experimental data (red data points). The diagonal line represents *D*_calc_=*D*_exp_. Only 19 out of 21 experimental RDCs are used in MSpin, because the two methoxy groups (26 and 28 in Figure 1) cannot be used for the analysis with this software.

In Figure 3, quality factors obtained from the MSpin calculations for the single best conformers of the 32 configurations are summarized. The *RRSSSSR* configuration has noticeably the best n/χ^2^ value, clearly identifying this configuration as the best fitting one out of the set of 32 single conformers. For most practical applications this result might be sufficient. However, the value for the best fitting configuration of n/χ^2^=0.093 also leads to the conclusion that the model is by far not sufficient to represent the experimental results. The overall quality factor is severely below 1 and more than 10 outliers disqualify the structural model. As the overall fit of experimental versus back‐calculated RDCs correlates well (see Figure 4), the most probable reason for the insufficient fulfillment of experimental data using the single conformer approach is the presence of flexibility in the RD‐1 molecule. As dynamics cannot be included in a single conformer approach, the extension to a multiple conformer analysis was attempted in the next step.

### Multiple conformer single alignment tensor SVD approach

From the existing approaches that use a combination of conformations, only the multiple conformer single alignment tensor SVD‐based fitting procedure is viable. The single‐tensor approach allows the calculation of RDCs based on a single alignment tensor for an ensemble of conformers of a given configuration. Clearly, fitting a single alignment tensor to a conformational ensemble is an approximation, which, however, successfully led to several configuration determinations in the past.[^1a^, ^21^]

**Figure 5:**
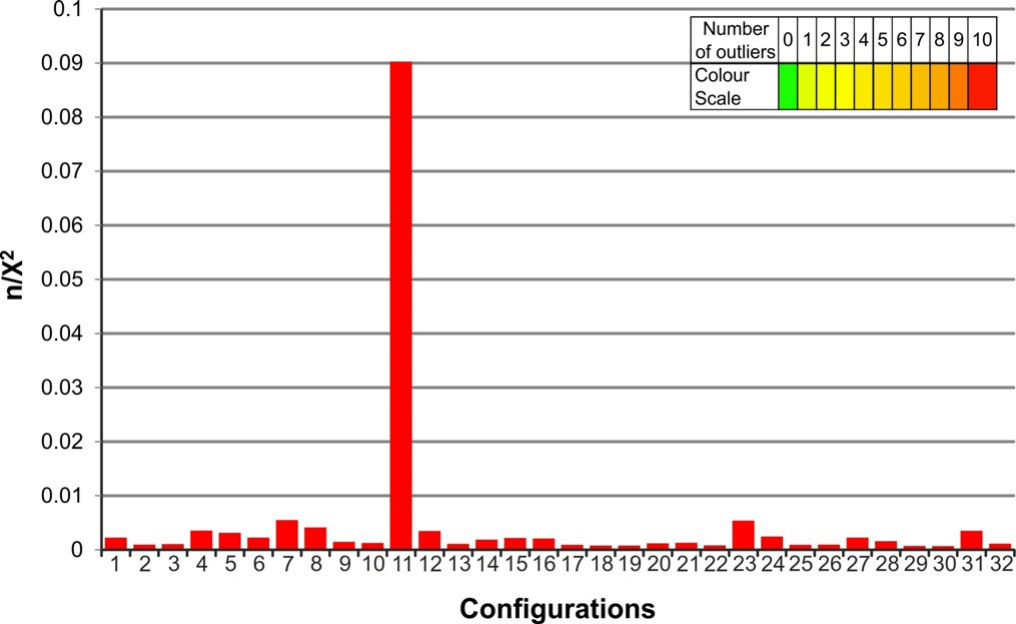
Multiple conformer single tensor fit quality factors n/χ^2^ calculated for all possible configurations of RD‐1 from multiple conformers. On the horizontal axis the 32 different configurations are listed using the numbering shown in Table 2. The color of the bar encodes the number of outliers from the measured RDCs values, as shown in the color scale on the upper right. Even the best static ensemble has 10 RDCs that do not comply with data within experimental errors.

**Figure 6:**
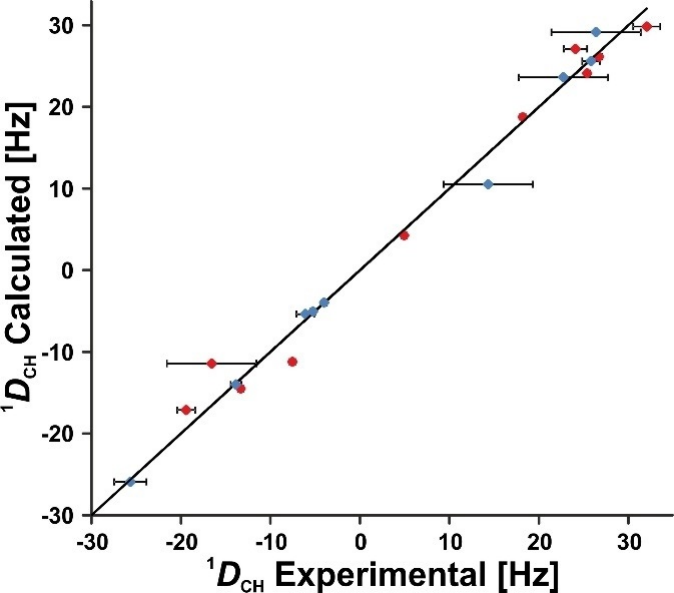
Plot of back‐calculated RDCs obtained with multiple conformer single tensor fit, against experimental RDCs for the best configuration 11 (*RRSSSSR*). Although the overall fit of experimental versus calculated data shows at first sight a good correlation, 10 out of the 19 RDCs are not within the range of maximum error estimates of the experimental data (red diamonds). The diagonal line represents the case of full correlation of experimental and calculated data. Only 19 out of 21 experimental RDCs are used in MSpin, because the two flexible methoxy groups (26 and 28 in Figure 1) have been excluded for the analysis with this software.

In our study, a number of different conformers was generated from a conformational search for each configuration with the software Macromodel. Using the default settings of the Monte Carlo‐based program with an OPLS 2005 force field, all conformations with an energy difference below 6.0 kcal mol^−1^ relative to the lowest energy structure were collected. In a first approach, all of these low‐energy conformers were combined to ensembles by weighing the population of each conformer according to its energy. The theoretical RDCs were then calculated using the single tensor approach. Resulting RDCs did not improve compared to the single conformer single alignment tensor fit and data are not shown. We then used the input ensemble of conformers for a fit where also the populations are optimized as the calculated energy‐differences might not represent the actual energies. With this approach, configuration 11 still has by far the best n/χ^2^ value (see Figure 5) and a look at Figure 6 shows the qualitatively good correlation of experimental and calculated data. However, the number of outliers remains high: 10 calculated RDCs are still outside the range of the maximum error estimates of the experimental RDCs. The result obtained for the best configuration 11 does practically not change compared to the single conformer approach. For this configuration, four different conformers are obtained with Maestro. These, however, only differ in the position of the methoxy groups that is, the core frame of the molecule is essentially the same for the different conformers and therefore very similar *D*_calc_ and consequently n/χ^2^ values are obtained. On the other hand, for some other configurations up to 16 different conformers are obtained by the Maestro software, partially with significant differences in the core frame. For example configuration 1 has 16 conformers and the SVD approach led to an improvement in n/χ^2^ from 0.0008 of a single conformer to approximately 0.002 for the multiple conformer fit. The multiple conformer single alignment tensor approach could therefore very slightly improve the overall quality of structural models, but consistency with experimental results could not be achieved.

### Dynamic simulation with orientational constraints (MDOC)

The program package COSMOS has a specialized protocol for time averaged molecular dynamics (MD) simulations with orientational constraints and also includes a molecular mechanics force field.[^6c^, ^22^] The orientation encoded in tensorial constraints derived from experimental RDC values is used as a factor for the generation of pseudo forces, which constrain the orientation of the molecule within the MD (for details of the approach see Tzvetkova et al.^[6c]^). For each MDOC step a full tensorial orientation is calculated and compared with the experimental data. Based on analytical solutions for the first and second derivative of the tensor with respect to the laboratory frame axes *x*, *y* and *z*, the MDOC run will rotate individual C−H bond vectors to improve the overall pseudo energy depending on the difference between each calculated and experimental RDC. For a successful MD run, different parameters have to be optimized, like the strength of pseudo forces (*k*) and the alignment scaling factor (*s*_AM_), which reduces the calculation time by avoiding unnecessary computation of isotropic tumbling of the molecule of interest.^[6c]^ In the optimized runs, we set *k* to 5.5.10^−4^ and *s*_AM_ to 4.10^−3^ to obtain calculated RDC values in the range of ^1^ *D*_CH_=−25.6 to 32.1 Hz (see Table 3 for the individual values). The MD runs were chosen to last for 80 ns in order to reach good convergence of the orientational constraints.

**Table 3:** Experimental versus computed values for configuration *RRSSSSR*.

	Experimental Data		MDOC	SVD (SC)	SVD (MC)
Couplings	*D*_exp_ [Hz]^[a]^	Δ*D*_exp_ [Hz]^[b]^	*D*_calc_ [Hz]^[c]^	*D*_calc_ [Hz]^[d]^	*D*_calc_ [Hz]^[e]^
C_10_H_10B_	14.3	5.0	12.7	10.4	10.5
C_10_H_10A_	22.7	5.0	21.9	23.7	23.6
C_21_H_21A_	−4.0	0.3	−4.2	−3.82	−4.0
C_21_H_21B_	18.2	0.3	18.1	18.8*	18.8*
C_5_H_5_	24.1	1.3	23.4	27.2*	27.1*
C_6_H_6_	25.8	1.0	25.3	25.7	25.6
C_22_H_22A_	25.4	0.3	25.1	24.3*	24.1*
C_22_H_22B_	−7.5	0.3	−7.5	−11.2*	−11.2*
C_4_H_4_	−13.8	0.6	−13.5	−13.9	−14.0
C_9_H_9B_	32.1	1.5	31.6	30*	29.9*
C_9_H_9A_	−13.3	0.3	−13.1	−14.4*	−14.5*
C_7_H_7B_	26.4	5.0	26.8	29.3	29.2
C_7_H_7A_	−16.6	5.0	−13.1	−11.3*	−11.4*
C_23_H_23_	26.7	0.3	26.6	26.2*	26.2*
CH_3‐28_	3.1	0.4	3.0		
CH_3‐26_	1.4	0.3	1.3		
C_2_H_2_	−25.6	1.8	−25.7	−26	−25.9
C_3_H_3_	5.0	0.3	5.2	4.2*	4.3*
C_19_H_19_	−5.2	0.3	−5.1	−5.4	−5.1
C_17_H_17_	−19.4	1.0	−19.3	−16.6*	−17.1*
C_16_H_16_	−6.1	1.0	−5.9	−5.4	−5.4

[a] Experimental values. [b] Experimental errors. [c] Averaged MDOC back‐calculated values. [d] Single conformer (SC) SVD back‐calculated values. [e] Multiple conformers (MC) SVD back‐calculated values. The values which do not reach the error range compared to the experimental data (1/χ^2^<1 outliers) are marked with an asterisk (*).

During the MDOC runs, at discrete time points as defined in the MDOC settings, the calculated ^1^ *D*_CH_ RDCs are written into an output file (here every 20 ps). At the evaluation of the MDOC runs, these ^1^ *D*_CH_ couplings are arithmetically averaged. The n/χ^2^ is then calculated from these averaged values neglecting the first nanosecond of the MDOC trajectory, which is needed for initial system equilibration. In addition, every 40 ps a geometry snapshot is saved as a control for the MD run.

**Figure 7:**
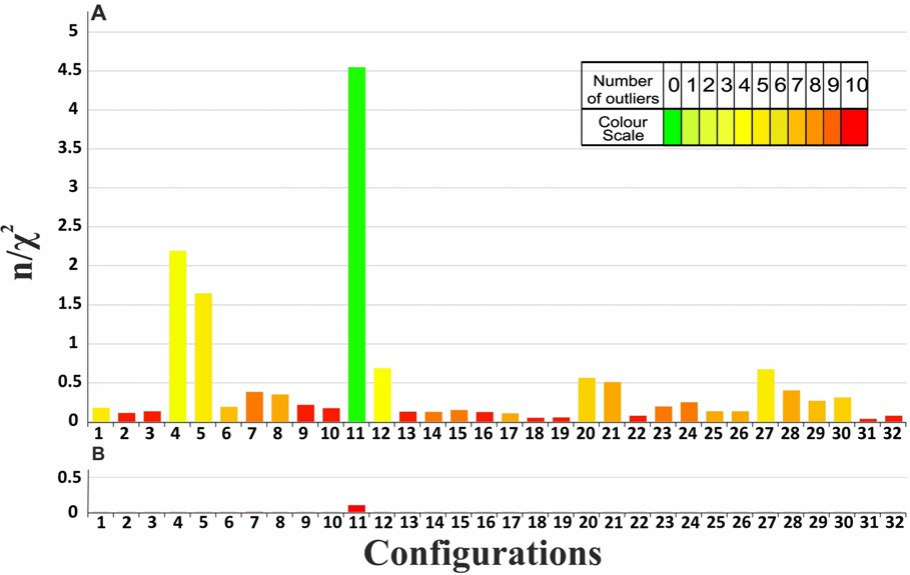
MDOC quality factors n/χ^2^ calculated for all possible configurations of RD‐1. On the horizontal axis the 32 different configurations are listed using the numbering of Table 2. The color of the bar encodes the number of outliers of the measured RDC values (i.e. 1/χ^2^<1). For comparison, the result of the multiple conformer single tensor fit is given with the same scale (same data as in Figure 5) emphasizing the much improved agreement with the data provided by MDOC (B).

**Figure 8:**
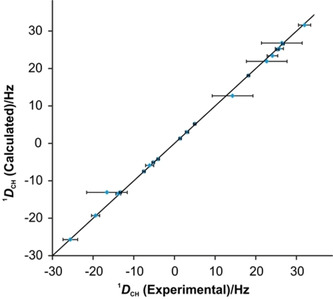
Plot of averaged back‐calculated RDCs obtained with MDOC, against experimental RDCs for the configuration 11 of RD‐1. All RDCs averaged over the MDOC run in COSMOS are inside the error range of the experimental data. The diagonal line represents the case of full correlation of experimental and calculated data.

The quality factors of the data obtained from COSMOS are summarized in Figure 7. While three configurations (4, 5, and 11) have overall quality factors n/χ^2^ above 1 and therefore potentially comply with experimental data, a detailed analysis reveals that only configuration 11 (*RRSSSSR*) fulfills all experimental constraints within the experimental errors while the other two matching structural models have two and five outliers, respectively. The comparison of measured and calculated RDCs, as shown in Figure 8, further supports the stereochemistry of configuration 11 (*RRSSSSR*) as the correct one.

Following the evaluation of ^1^ *J*_CH_ coupling constants as described above, amine inversion was prevented during MDOC runs by fixed distances in the amine surrounding as described in the SI. Without the additional distance constraints amine inversion occurs and configurations differing only by an inversion of N8 are virtually indistinguishable (see SI), leading, however, to very similar results with just slightly reduced quality factors.

### X‐ray data

**Figure 9:**
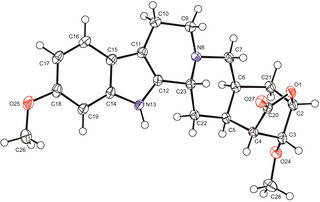
Crystal structure of RD‐1. Non‐H atoms are represented by displacement ellipsoids drawn at 50 % probability level.

In parallel to the NMR efforts, we were able to determine the crystal structure of RD‐1. The structure analysis allowed determining the configuration of RD‐1 as configuration 11 from NMR‐based analysis, which is in full agreement with the RDC‐based NMR analysis of all seven stereocenters. Based on the presence of anomalous scatterers oxygen and nitrogen, the absolute configuration of RD‐1 could be unambiguously assigned as *C2R, C3R, C4S, C5S, C6S, N8S, C23R* (numbers refer to X‐ray structure labelling scheme shown in Figure 9). The result is supported by a Flack *x* parameter of 0.02 (13).^[23]^

### Comparison of NMR‐based approaches

Out of previously reported direct fitting approaches for the determination of conformational ensembles based on residual dipolar couplings, to the knowledge of the authors only two approaches are practically feasible for medium‐sized organic molecules like RD‐1 with a limited set of RDCs. All other approaches are either too complex to be applied to this class of molecules, as for example, methods based on the mean field additive potential principle,^[24]^ or too few RDCs are accessible for a reliable fit, as is the case for the multiple conformer multiple alignment tensor fit. The two remaining approaches used here are both based on fitting a single alignment tensor either to a single rigid conformer or to a set of selected conformers using singular value decomposition (SVD). In both cases an ensemble of possible conformers, usually representing lowest‐energy structures, is preselected typically based on ab initio calculations or other computer aided structure elucidation approaches.

In contrast to these direct fitting methods, constrained molecular dynamics (MD) simulations are used to optimize a single structure (e.g. via simulated annealing) or an ensemble of structures that best fit experimental results. Several implementations have been reported based on an approximated alignment tensor as initial input.^[25]^ An alignment tensor for a flexible molecule, however, is *per se* ill‐defined and the recently published MDOC approach seems far more appropriate in this case.[^6c^, ^7a^]

Out of the three approaches applied to RD‐1, both alignment tensor approaches led to basically identical results: from the preselected conformers of all possible 32 relative configurations clearly the correct one, which was simultaneously identified by X‐ray diffraction, fitted the data best, demonstrating again the enormous potential of RDCs for structure and in particular configuration determination. The molecule with seven stereogenic centers represents a complex structure that at first sight might be considered relatively rigid. The configuration and dynamics at the amine N8 could not be determined by using NOE and ^3^ *J‐*coupling data alone. Based on the measurement of ^1^ *J*_CH_ constants (see Table 1) and using the Perlin effect, fast inversion at N8 can be excluded to a large extend. However, the preselected conformers did not allow producing a structural ensemble that fully agreed with experimental data using the SVD fitting approach. Even in the best case 10 out of 19 RDCs were outside the error margins of the experiment. Apparently, additional conformers would have to be taken into account that were not part of a standard conformational search procedure, and potentially multiple individual alignment tensors would have been needed. A certain deviation of RDCs is expected from vibrational correction,^[26]^ but the large differences cannot fully be explained this way.

**Figure 10:**
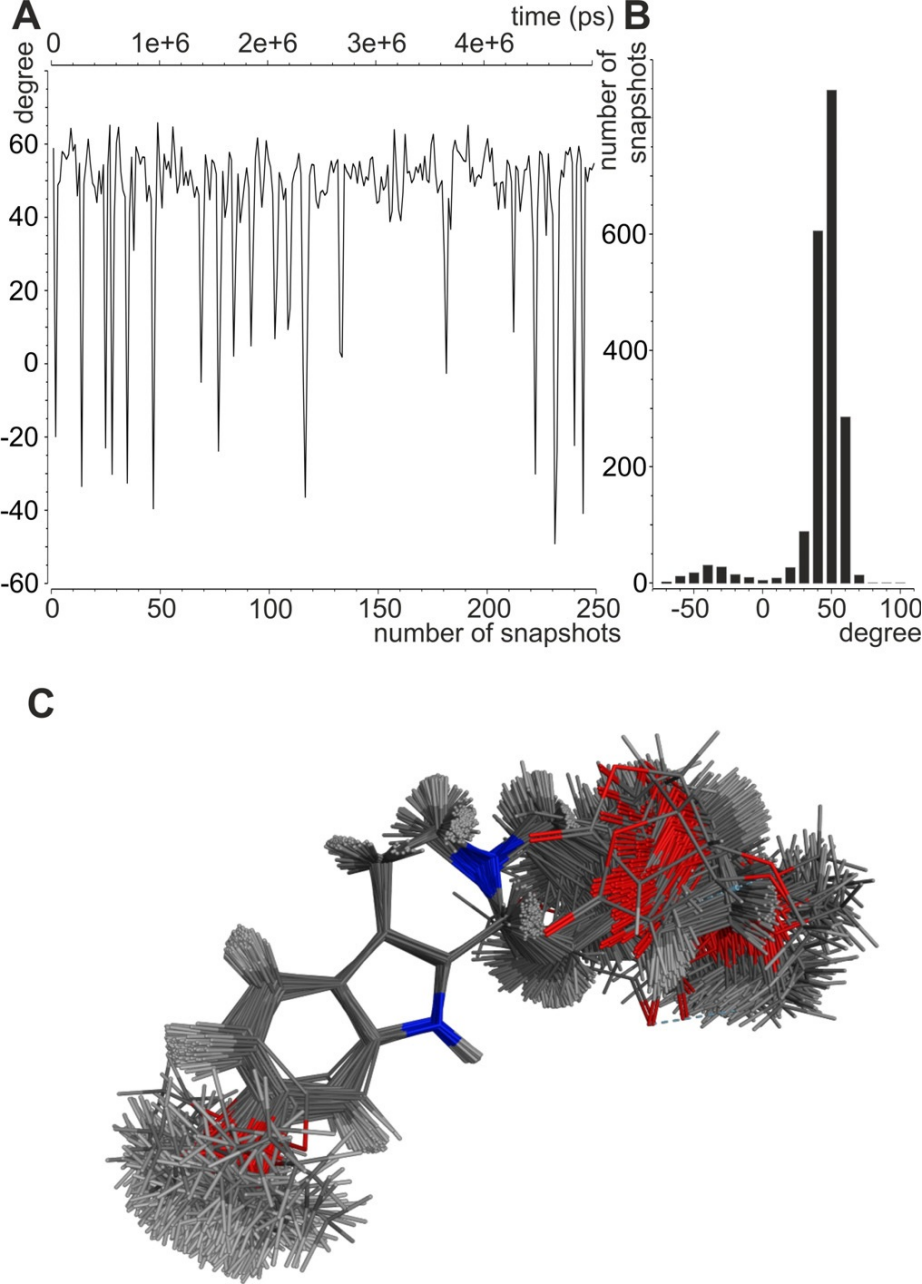
Minor conformation from experimental RDC constraints. (A, B) The dihedral angle H_10A_‐C_10_‐C_9_‐H_9A_ is indicative for at least one minor conformation with a population of roughly 10 %. A graph with the evolution of the angle over a part of the MDOC run (A) as well as the population of the angles over the entire MDOC run is shown (B). (C) Visualization of the structural ensemble of the correct relative configuration of RD‐1 from 250 individual conformations from the MDOC run. Structures are overlaid at the indole ring.

The MDOC approach, in contrast, has no pre‐assumptions concerning the conformational space and the correct structure results in an orientational and conformational ensemble that fully reproduces the experimental data. The resulting structural ensemble reveals the large dynamics of RD‐1 (Figure 10), partly caused by vibrational motions, but also by distinct conformational changes, as visualized by the time‐dependence and distribution of the example dihedral angle H_10A_‐C_10_‐C_9_‐H_9A_. According to the dihedral distribution of the MDOC ensemble up to 10 % population can be attributed to at least one minor conformation which is not *per se* obvious in the NMR experimental data. The few accessible scalar coupling constants did not allow the detection of such a minor conformer, but line broadening in the C‐ring has been observed, corroborating qualitatively the determined flexibility and the existence of a minor conformer. As the corresponding conformer is missing in the preselection of the alignment tensor fitting approaches, it explains the inability of both single and multiple conformer fits to reproduce the experimental RDCs.

As the full accessible conformational space is only restricted by RDC constraints in the MDOC approach, the ability to discriminate the different relative configurations must necessarily be reduced. Still, if both n/χ^2^ values and the number of outliers are taken into account, the correct configuration 11 is unambiguously determined.

## Conclusions

In summary, we show with the example of a highly complex molecule that RDCs represent a valuable tool for the determination of the conformation and configuration of even very complex organic molecules and demonstrate the abilities of state‐of‐the‐art data interpretation approaches for the distinction of relative configurations and the determination of a structural ensemble that fulfills all experimental constraints within error margins. The configuration of seven stereogenic centers in a partially flexible molecule (RD‐1) could be determined, including the stereochemistry of an amine. This was not possible using only standard NMR data (NOEs and scalar couplings). For a partially flexible molecule as shown here, the fast SVD approach with preselected conformers for each configuration led to a clear discrimination of the correct versus wrong configurations of RD‐1, corroborating the CASE‐3D analysis approach.^[27]^ With a limited set of RDCs this approach is certainly the way to go for a simple configurational analysis. However, experimental data could not be reproduced within their error ranges, indicating a clear lack in the coverage of conformational space. The latter was straightforwardly achieved by the so‐called MDOC (molecular dynamics with orientational constraints) approach, which resulted in a valid conformational ensemble and also provided a clear distinction of diastereomers unbiased by a lowest‐energy preselection of conformers. In essence, all three RDC‐based approaches provide a clear and correct determination of configuration of the complex molecule RD‐1, proven for six out of the seven stereocenters by X‐ray crystallography (except the amine), and the recently introduced MDOC approach in addition leads to a valid orientational and conformational ensemble, demonstrating the unique power of anisotropic NMR parameters and the high level of state‐of‐the‐art data interpretation.

## Experimental Section

**Sample preparation**: The isotropic sample of RD‐1 was prepared by dissolving 2.8 mg in 0.5 mL of [D_6_]DMSO leading to a final concentration of 14.7 m. The partially aligned sample in PAN/[D_6_]DMSO contained a dry polymer stick of cross‐linked PAN placed inside the Kalrez® tubing of the stretching apparatus with 300 μL [D_6_]DMSO and 10 mg of compound leading to an approximate concentration of 87.2 mm. A dry PAN polymer stick of 3 mm diameter irradiated with accelerated electrons (200 kGy) was used.^[12i]^

**NMR spectra for the assignment in isotropic phase**: All NMR measurements (^1^H‐1D, HSQC, COSY, ROESY and HMBC) for the assignment of RD‐1 in [D_6_]DMSO were recorded at 26 °C on a Bruker 500 MHz Avance III spectrometer (500.09 MHz for ^1^H and 125.75 MHz for ^13^C) equipped with a 5 mm BBFO probe head with actively shielded *z‐*gradients. The ^1^H NMR spectrum was acquired by using 64k data points at a spectral width of 12 kHz, and a 1.5 s repetition delay. 2D ^1^H,^13^C‐correlation spectra were recorded with 2k data points in the ^1^H dimension and 128 points in the ^13^C dimension. 2D ^1^H,^1^H‐correlation spectra were recorded with 2k data points and 128 points in the indirect dimension. The repetition delay for the 2D experiments was 1 s.

**NMR spectra for the RDC measurements**: The NMR spectra were recorded on Bruker 800 MHz Avance III HD spectrometer equipped with a 5 mm CPTCI inversely detected ^1^H,^13^C,^15^N triple resonance cryogenically cooled probe with actively shielded *z‐*gradients and frequencies of 800.16 MHz for proton, 201.20 MHz for carbon and 122.83 MHz for ^2^H. The temperature was controlled with a Bruker SmartVT‐unit to be 26 °C throughout all experiments. To assess the introduced alignment using the stretching device, a deuterium spectrum was recorded leading to a quadrupolar splitting of Δν_Q_=6.3 Hz. The homogeneity of the alignment media was controlled by a deuterium imaging experiment.^[28]^ The residual dipolar coupling values were obtained from CLIP‐HSQC^[15]^ and P.E.HSQC^[16]^ experiments under both isotropic and anisotropic conditions. A P.E.HSQC spectrum was measured in addition in order to compare and confirm the residual dipolar couplings. All 2D spectra were acquired with 32k(^1^H)*512(^13^C) data points, unless stated otherwise. The repetition delay was set to 1 s. The ^1^H,^13^C‐CLIP‐HSQC spectrum in isotropic condition was acquired with a 64k(^1^H)*512(^13^C) data matrix, while the, P.E.HSQC in the isotropic case had 1.5k points in the indirect dimension. All spectra were processed using the software Topspin 3.2 and were apodized by a 90° shifted sine squared window function for ^13^C and for ^1^H, with prediction of 512 points and zero filling up to 2k points in the directly acquired dimension. The couplings were measured by superimposing the left side of the split signals with the right side of the same signal from a second copy of the same row of the 2D experiment. The experimental errors were determined as maximum error estimates following the procedure described in [17].

**Generation of chemical structures for all configurations**: The initial three‐dimensional structure of each possible configuration was built with CORINA.^[19]^ All trial structures were energy minimized using Schrödinger Release 2014‐2 (Maestro, Schrödinger, LLC, New York, NY, 2016). The conformational search for each configuration was realized with MacroModel (Schrödinger Release 2014‐2 Macromodel, Schrödinger, LLC, New York, NY, 2016). OPLS 2005 was used as force field and the conformational research was done in Low‐Frequency‐Mode with default parameters in water.

**MSpin: Fitting experimental RDCs using singular value decomposition**: The fitting procedure of the experimental RDC data was performed by using the MSpin program.^[5]^ 19 experimentally determined ^1^ *D*_CH_ couplings from RD‐1 and the coordinate files of 32 possible configurations were given as input data. The alignment tensors were determined using singular value decomposition (SVD). The fit between experimental and back‐calculated RDCs is given by default with the Cornilescu quality factor Q^[20]^ [Eq. 1]:

(1) $$Q=\sqrt{\frac{{\sum_{i}\left(D_{calc}^{i}-D_{exp}^{i}\right)}^{2}}{{\sum_{i}\left(D_{exp}^{i}\right)}^{2}}}$$

For our study the fit between experimental and back‐calculated RDCs was expressed with the value n/χ^2^ in order to have a uniform quality criterion with the MDOC results^[6c]^ where n is the number of experimentally obtained RDC values and χ^2^ is defined as [Eq. 2, Eq. 3]:

(2) $$\chi_{i}^{2}={\left(\frac{D_{calc}^{i}-D_{exp}^{i}}{\Delta D_{exp}^{i}}\right)}^{2}$$

(3) $$\frac{n}{\chi^{2}}=n\;\sum_{i=1}^{n}\frac{1}{\chi_{i}^{2}}$$

where i runs over all measured RDC data, *D_calc_* and *D_exp_* are the back‐calculated and the experimental values, respectively, and Δ*D* ^i^_exp_ are the experimental errors of each *D*_exp_ given as maximum error estimates.^[17]^

**Fitting experimental RDCs using single tensor fit with multiple conformers**: For this approach the MSpin program has been used. A common coordinate system for all conformations is determined by the superposition of the different geometries. The populations of conformers generated for a given configuration have been both weighted with the energies provided by Maestro (Maestro, Schrödinger, LLC, New York, NY, 2016) using the molecular force‐field OPLS 2005 in Macromodel (Schrödinger Release 2014‐2: Macromodel, Schrödinger, LLC, New York, NY, 2016) or directly optimized within the fitting procedure. Data have been fitted using the single‐tensor procedure and back‐calculated RDCs (*D*_calc_) have been compared to the experimental data (*D*_exp_) allowing the determination of the quality factor n/χ^2^.

**MDOC**: The MDOC simulations were performed using COSMOS 6.0 with the COSMOS force field.^[6b]^ Each MDOC simulation was run for 80 ns with 160 million steps. Snapshot coordinates were saved every 40 ps resulting in 2000 snapshots for the flexibility analysis in MSpin. Different distance types were fixed during the MD simulations: one bond C−H distances and the distances between the carbons around the amine in order to avoid unphysical inversion of the amine. The experimental RDC data were used to determine the relative configuration of RD‐1 as orientational constraints. For the MDOC run the dipolar couplings were scaled with a scaling factor 4.0×10^−3^ as previously described ^6c^. The optimal value for the pseudo force constant for RD‐1 was optimized to 5.5×10^−4^ following the procedure described in [6c, 7a]. The temperature is monitored via a thermal bath in order to control the behavior of the molecule during the MD; it is set to 300 K. The COSMOS output files with the back‐calculated RDC values were evaluated via home‐written scripts in Matlab (The MathWorks, Inc.). The first nanosecond of the MD runs were excluded to avoid equilibration artefacts and all further sampled values were used to generate arithmetic averaging of RDCs. Subsequently, averaged RDC values obtained from 4000 snapshots were used to determine quality factors n/χ^2^ for all 32 relative configurations as shown in Figure 7.

**X‐ray diffraction**: Crystals of the reserpine derivative RD‐1 were obtained from a solution of RD‐1 in tetrahydrofurane by slow evaporation of the solvent at room temperature. Diffraction data were collected at 100 K on a Bruker AXS MicroStar diffractometer using a SMART 6000 CCD detector on a three‐circle platform goniometer with Cu(K_α_) radiation (*λ*=1.54178 Å) from a microfocus rotating anode generator equipped with Incoatec multilayer optics. 16 ω‐scans at different ϕ‐positions were performed to ensure appropriate data redundancy (5.9, Friedel pairs not merged). The crystal structure was solved by dual space‐recycling methods and refined based on full‐matrix least‐squares on F2 using the SHELXTL program suite (Sheldrick GM (2001)). Anisotropic displacement parameters were used for all non‐hydrogen atoms. Hydrogen atoms were located in a DF map and refined in idealized positions using a riding model. The absolute structure was determined based on the anomalous scatterers present (O and N). For the *C2R, C3R, C4S, C5S, C6S, N8S, C23R* diastereomer the Flack *x* parameter refined to 0.02(13). In the crystal structure the amine is only present in one configuration (*S*). This prevalence is, however, i.e. based on data collected on one crystal only and might be solid‐state‐driven, it is not necessarily reflecting the distribution of the N8*R* and N8*S* diastereomers in solution.

Deposition number 19997972 contains the supplementary crystallographic data for this paper. These data are provided free of charge by the joint Cambridge Crystallographic Data Centre and Fachinformationszentrum Karlsruhe Access Structures service.

## Conflict of interest

The authors declare no conflict of interest.

## Supporting information

As a service to our authors and readers, this journal provides supporting information supplied by the authors. Such materials are peer reviewed and may be re‐organized for online delivery, but are not copy‐edited or typeset. Technical support issues arising from supporting information (other than missing files) should be addressed to the authors.

Supplementary
