# Supplementary material for: Determination of Configuration and Conformation of a Reserpine Derivative with Seven Stereogenic Centers Using Molecular Dynamics with RDC‐Derived Tensorial Constraints
Source: Chemistry. 2020 Oct 1;26(63):14435–44. doi: 10.1002/chem.202002642 (PMC7702126; doi:10.1002/chem.202002642)
Supplement: Supplementary file 1 — Supplementary [file CHEM-26-14435-s001.pdf]

Chemistry–A European Journal

Supporting Information

Determination of Configuration and Conformation of a Reserpine Derivative with Seven Stereogenic Centers Using Molecular Dynamics with RDC-Derived Tensorial Constraints**

Emine Sager,[a, b] Pavleta Tzvetkova,*[c] Alvar D. Gossert,[b, d] Philippe Piechon,[b] and Burkhard Luy*[a, c]

Author Contributions

B.L. Conceptualization: Equal; Project administration: Lead; Supervision: Equal; Validation: Equal; Writing - Original Draft: Equal
E.S. Data curation: Lead; Investigation: Lead; Writing - Original Draft: Lead
P.T. Conceptualization: Equal; Data curation: Supporting; Investigation: Equal; Supervision: Lead; Writing - Original Draft: Supporting
A.G. Conceptualization: Supporting; Data curation: Supporting; Investigation: Supporting; Supervision: Supporting; Writing - Review & Editing: Equal
P.P. Data curation: Supporting; Validation: Supporting; Writing - Original Draft: Supporting.

Table of Contents

Structure of reserpine 2

Experimental data for Reserpine Derivate 1 (RD-1) 2

MDOC results without the fixation of the amine 9

Structure of RD-1 with the fixation of the amine in order to avoid the inversion during the MDOC 9

Coordinates of RD-1 in pdb file format 10

Coordinates of RD-1 in coo-file format for the correct configuration 12

Data file for COSMOS MD simulation (.cod) for RD-1 14

Data file for MSpin SVD fitting calculation for RD-1 16

MDOC run Project file (.cos) for RD-1 17

MSpin and COSMOS results for all configurations of RD-1 24

Comparison of COSMOS *vs.* MSpin outliers 26

X-Ray: Crystallisation process 26

Structure of reserpine

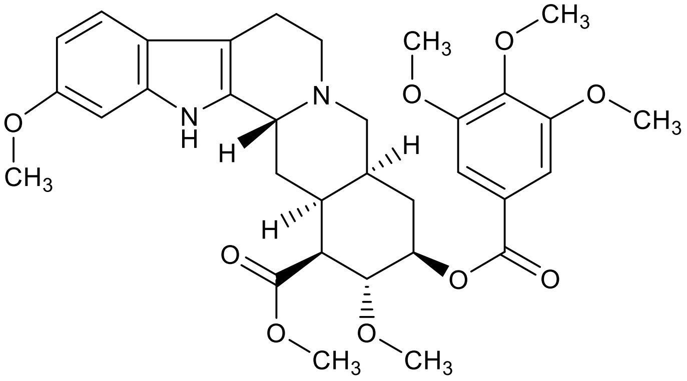

Figure S1: Structure of reserpine

Experimental data for Reserpine Derivate 1 (RD-1)

NMR spectra of RD-1

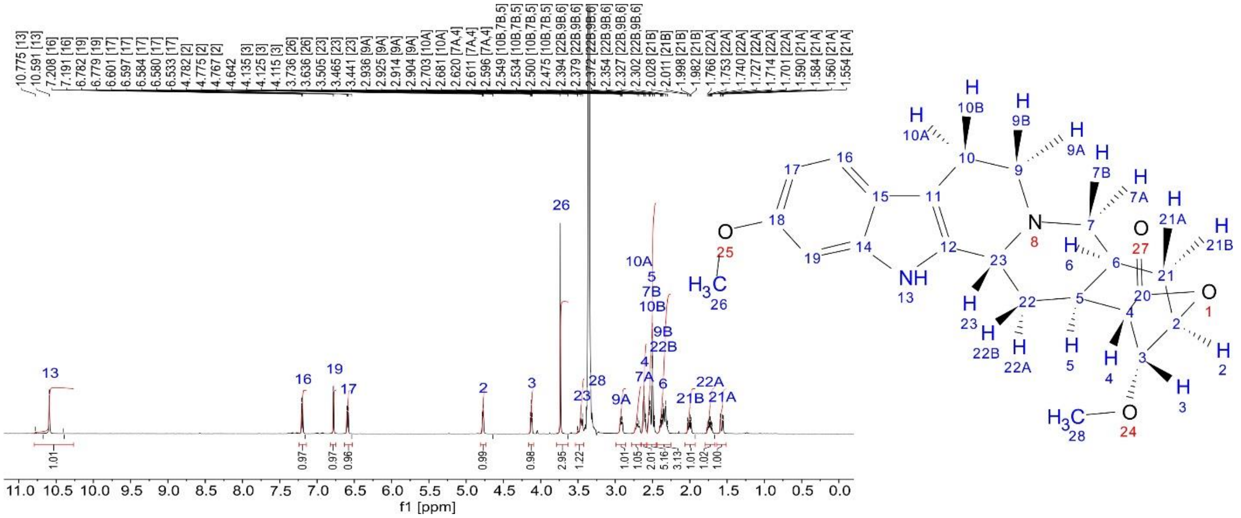

Figure S2: ^{1}H NMR spectrum with assignment of RD-1 in DMSO-d_6.

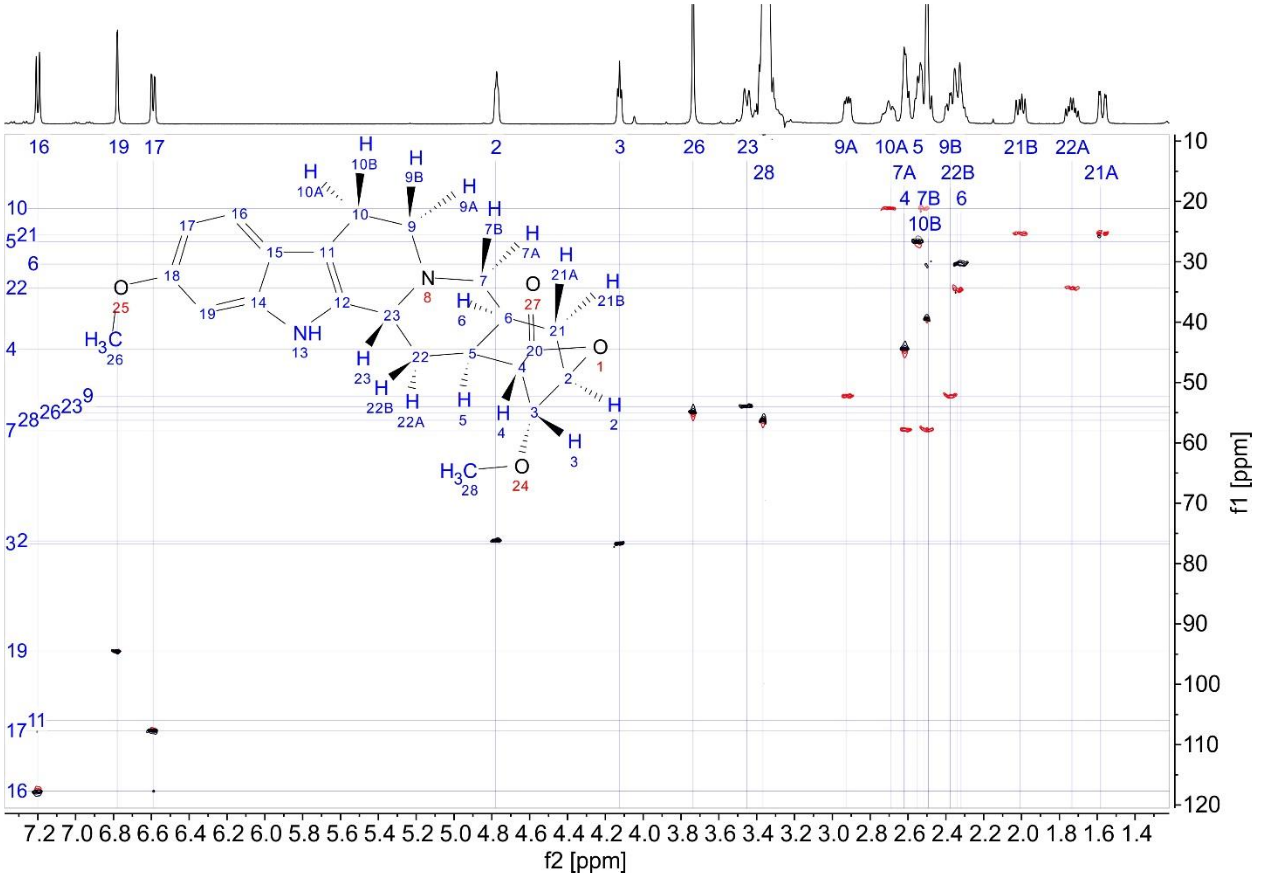

Figure S31: ^{1}H,^{13}C-Edited-HSQC spectrum with assignment of RD-1 in DMSO-d_6.

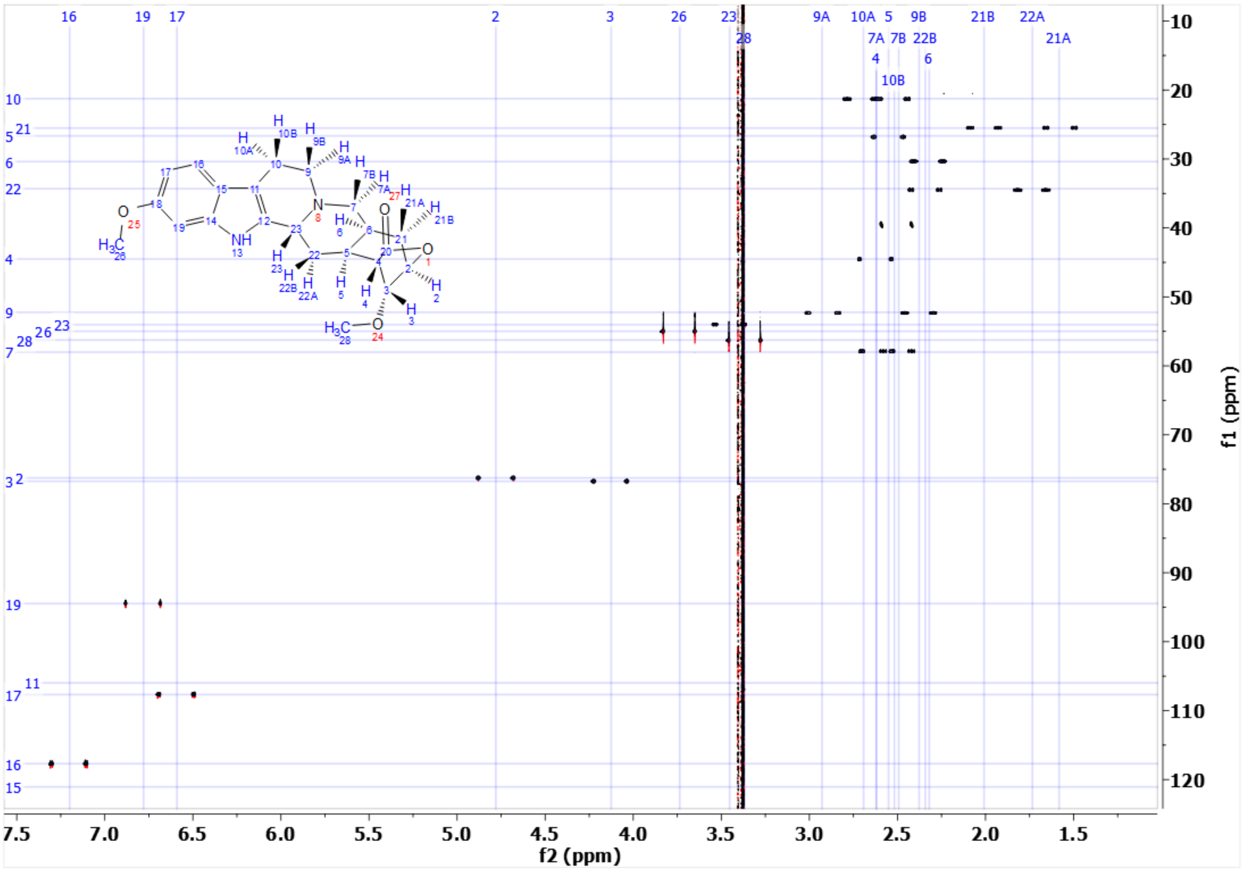

***Figure S4**:* $^{1}H,^{13}C$-CLIP-HSQC spectrum with assignment of RD-1 in DMSO-d_6

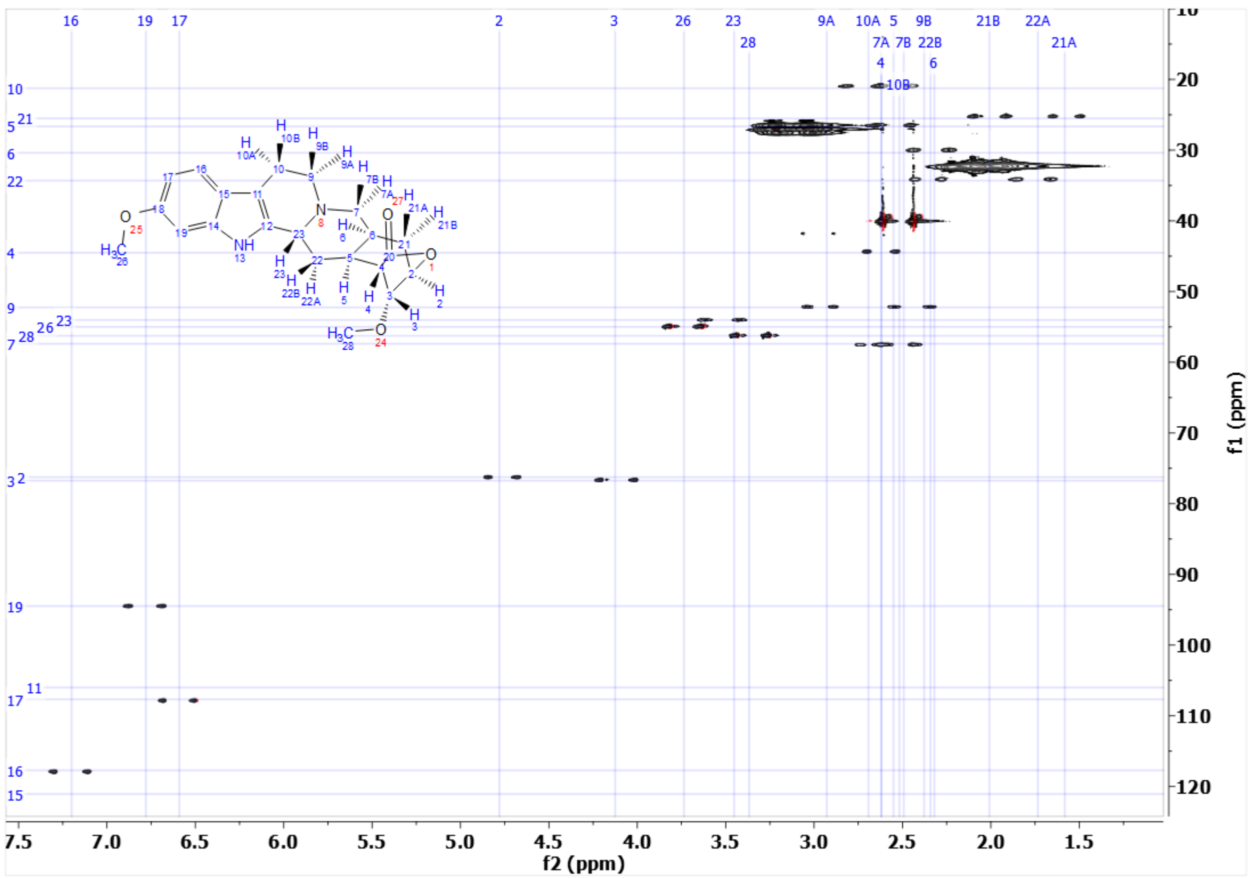

***Figure S5**:* $^{1}H,^{13}C$-CLIP-HSQC spectrum with assignment of RD-1 in PAN/DMSO-d_6

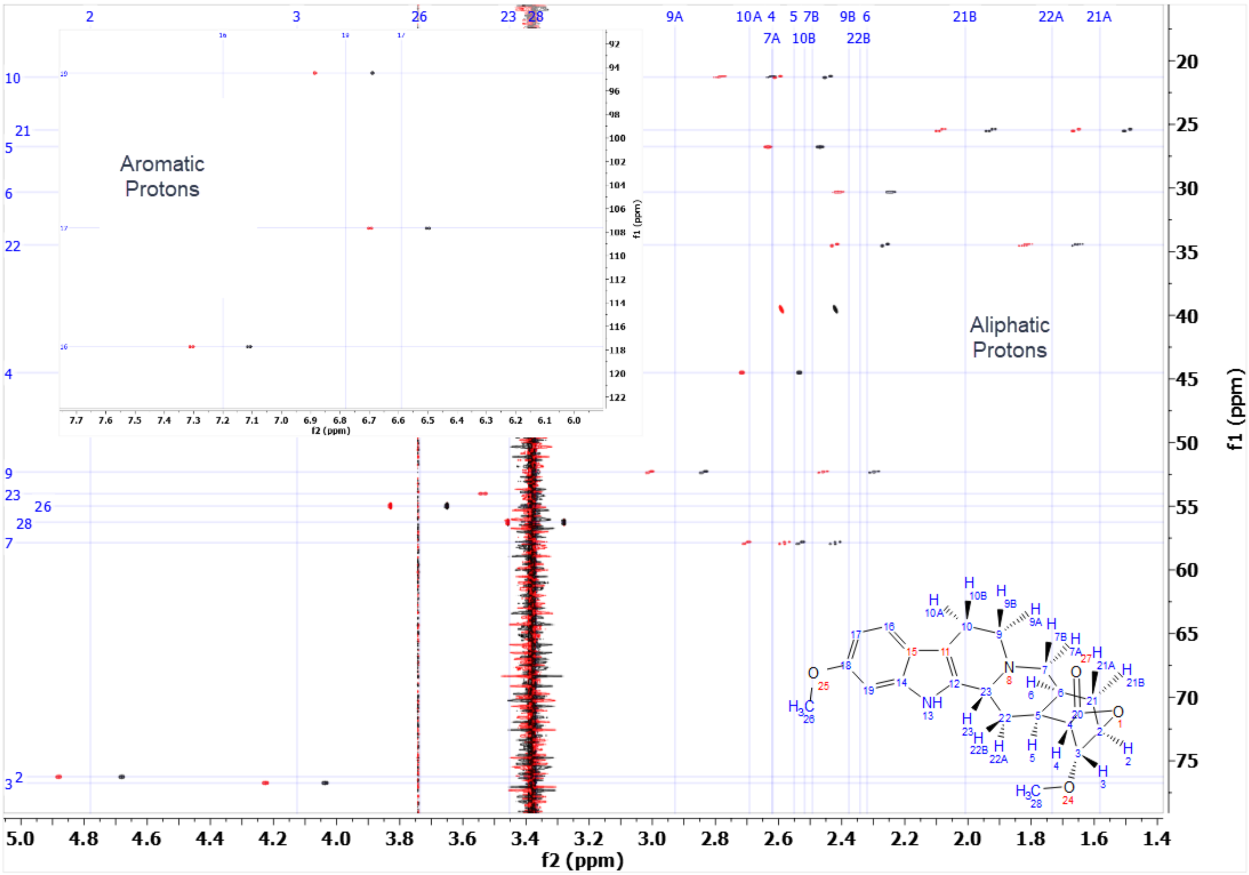

Figure S6: $^{1}H,^{13}C$-P.E.HSQC spectrum with assignment of RD-1 in DMSO-d_6

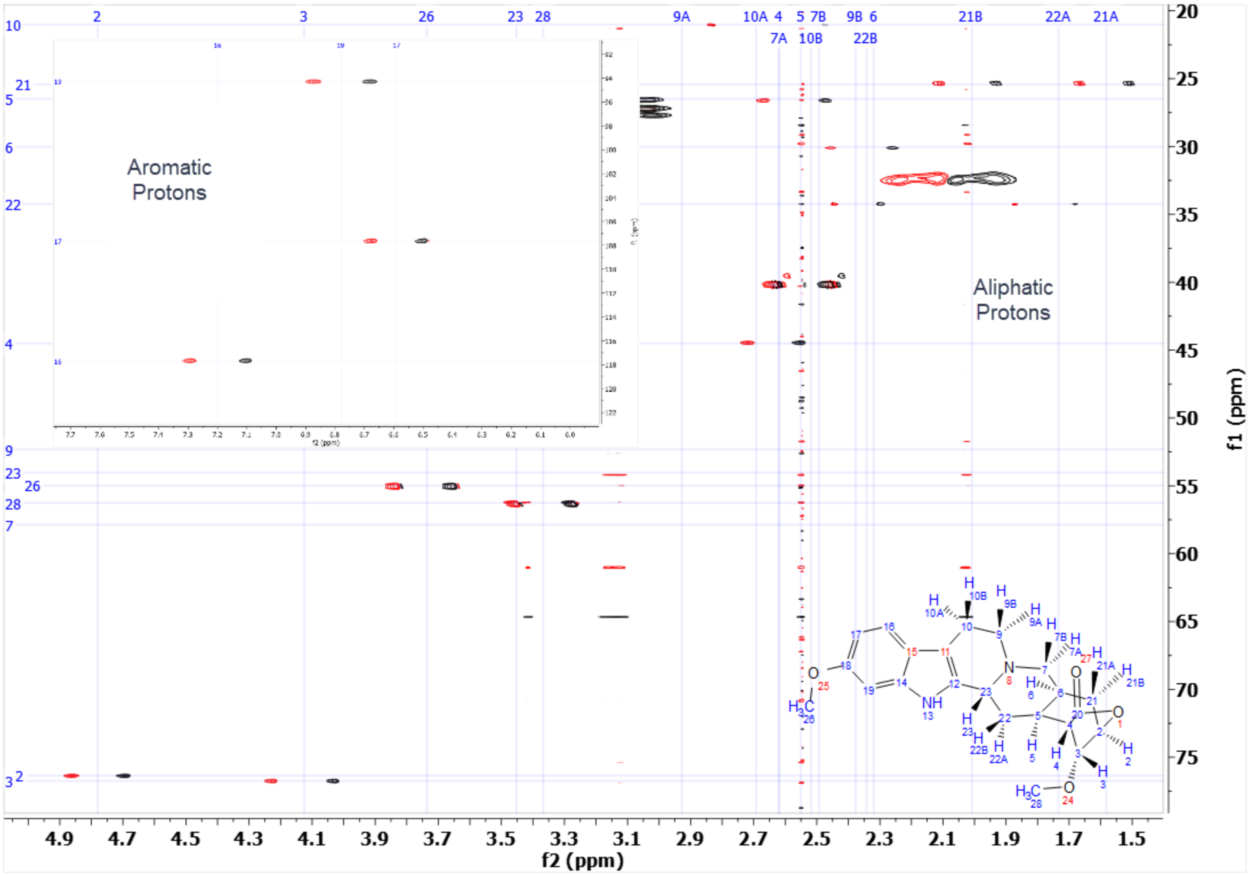

***Figure S7**:* ^{1}H,^{13}C-P.E.HSQC spectrum with assignment of RD-1 in PAN/DMSO-d_6

Table S1. Experimental data for RD-1 in DMSO-d_6

	δ_{1H}/[ppm]	*Multiplicity* J_{HH}/[Hz]	δ_{13C}/[ppm]	Observed NOEs with
C_2-H_2	4.77	t[3.7]	76.3	H_3, H_{21B}, H_{21A}
C_3-H_3	4.13	t[5.0]	76.8	H_2, H_3
C_4-H_4	2.62	m	44.5	H_3
C_5-H_5	2.55	m	26.7	
C_6-H_6	2.32	m	30.4	
C_7-H_{7A}	2.62	m	57.8	H_{21A}
C_7-H_{7B}	2.50	m	57.8	
C_9-H_{9A}	2.93	dd[5.1, 10.8]	52.2	H_{9B}
C_9-H_{9B}	2.38	m	52.2	H_{9A}, H_{23}
C_{10}-H_{10A}	2.69	m	21.2	H_{10B}
C_{10}-H_{10B}	2.52	m	21.2	H_{10A}
C_{11}			106.0	
C_{12}			134.8	
NH_{13}	10.59	s		H_{19}, H_{22A}, H_{22B}f
C_{14}			136.7	
C_{15}			121.1	
C_{16}-H_{16}	7.20	d[8.5]	117.7	H_{10B}, H_{17}
C_{17}-H_{17}	6.59	dd[1.8, 8.4]	107.7	H_{26}
C_{18}			154.9	
C_{19}-H_{19}	6.78	d[1.5]	94.5	H_{26}
C_{20}			177.6	
C_{21}-H_{21A}	1.57	dd[3.4, 15.0]	25.3	H_{7A}, H_{21B}
C_{21}-H_{21B}	2.00	dd[8.4, 15.0]	25.3	
C_{22}-H_{22A}	1.73	td[6.9, 13.2]	34.3	H_{22B}
C_{22}-H_{22B}	2.34	m	34.3	H_{22A}
C_{23}-H_{23}	3.45	d[12.0]	54.0	H_{9B}
C_{26}-H_{26}	3.74	s	55.0	H_{17}, H_{19}
C_{28}-H_{28}	3.36	s	56.3	

MDOC results without the fixation of the amine

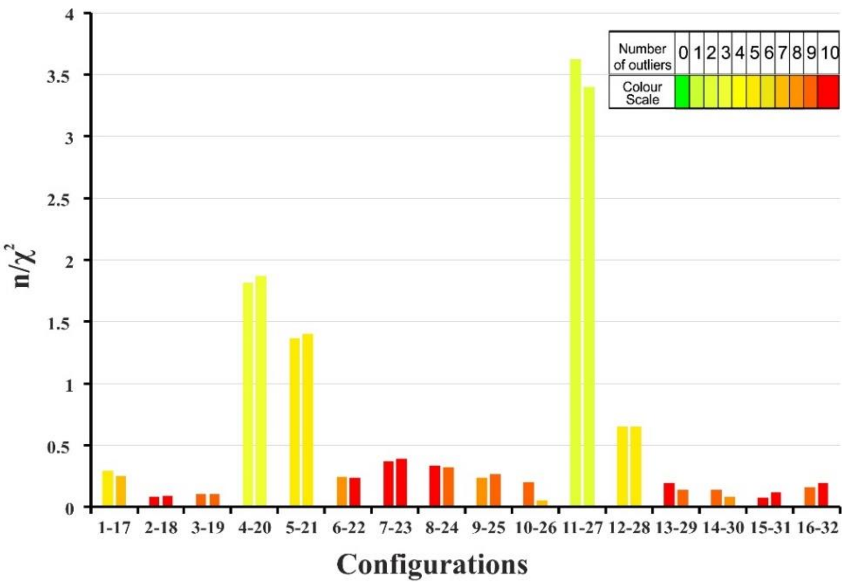

Figure S7: COSMOS quality factors n/χ^2 calculated for all possible relative configurations of RD-1.
On the horizontal axis, the 32 different configurations are listed using the numbering of Table 2. The colour of the bar encodes the number of outliers of the measured RDCs values taken from 1/χ^2 values below 1.

Structure of RD-1 with the fixation of the amine in order to avoid the inversion during the MDOC

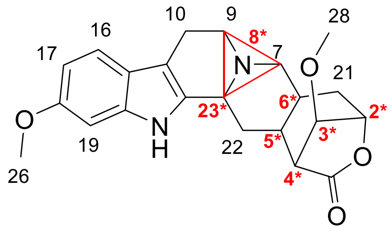

Figure S8: Structure of RD-1
In order to avoid an inversion of the amine during the MDOC run, the distances shown as red lines in the following Figure have been fixed.

Coordinates of RD-1 in pdb file format

The coordinates of the correct configuration of RD-1 (configuration 11) in pdb-file format generated as an output from the program CS Chem3D.

```
REMARK This PDB file was created by CS Chem3D.
HETATM    1  C           1      -1.073   1.184   0.007                         C
HETATM    2  C           1       0.089   0.266   0.284                         C
HETATM    3  C           1      -1.081   2.472   0.382                         C
HETATM    4  N           1      -2.186   0.949  -0.563                         N
HETATM    5  N           1       1.284   1.033   0.625                         N
HETATM    6  C           1       0.369  -0.650  -0.913                         C
HETATM    7  C           1      -2.257   2.980  -0.005                         C
HETATM    8  C           1       0.055   3.127   1.099                         C
HETATM    9  C           1      -2.921   1.982  -0.597                         C
HETATM   10  C           1       2.352   0.139   1.046                         C
HETATM   11  C           1       1.001   2.042   1.642                         C
HETATM   12  C           1       1.575  -1.584  -0.717                         C
HETATM   13  C           1      -2.808   4.192   0.100                         C
HETATM   14  C           1      -4.146   2.169  -1.096                         C
HETATM   15  C           1       2.765  -0.852  -0.064                         C
HETATM   16  C           1       1.251  -2.927  -0.030                         C
HETATM   17  C           1      -4.042   4.368  -0.407                         C
HETATM   18  C           1      -4.740   3.379  -1.014                         C
HETATM   19  C           1       3.854  -1.841   0.416                         C
HETATM   20  C           1       2.445  -3.881  -0.100                         C
HETATM   21  C           1       1.146  -2.801   1.481                         C
HETATM   22  O           1      -6.003   3.515  -1.542                         O
HETATM   23  C           1       3.299  -3.173   0.939                         C
HETATM   24  O           1       3.002  -3.959  -1.379                         O
HETATM   25  O           1       2.406  -2.921   1.986                         O
HETATM   26  O           1       0.141  -2.620   2.131                         O
HETATM   27  C           1      -6.658   4.757  -1.432                         C
HETATM   28  C           1       4.026  -4.926  -1.473                         C
HETATM   29  H           1      -0.227  -0.352   1.157                         H
HETATM   30  H           1       3.221  -0.202  -0.858                         H
HETATM   31  H           1       1.882  -1.864  -1.758                         H
HETATM   32  H           1      -2.467   0.021  -0.955                         H
HETATM   33  H           1       0.570   0.015  -1.789                         H
HETATM   34  H           1      -0.535  -1.250  -1.170                         H
HETATM   35  H           1      -0.324   3.769   1.928                         H
HETATM   36  H           1       0.593   3.776   0.368                         H
HETATM   37  H           1       3.256   0.739   1.315                         H
HETATM   38  H           1       2.055  -0.393   1.976                         H
HETATM   39  H           1       1.940   2.547   1.975                         H
HETATM   40  H           1       0.552   1.564   2.546                         H
HETATM   41  H           1      -2.266   5.018   0.587                         H
HETATM   42  H           1      -4.670   1.326  -1.576                         H
HETATM   43  H           1       0.336  -3.402  -0.452                         H
HETATM   44  H           1      -4.469   5.378  -0.304                         H
HETATM   45  H           1       4.489  -1.365   1.200                         H
HETATM   46  H           1       4.548  -2.060  -0.430                         H
HETATM   47  H           1       2.140  -4.900   0.239                         H
HETATM   48  H           1       4.115  -3.828   1.321                         H
HETATM   49  H           1      -7.661   4.655  -1.904                         H
HETATM   50  H           1      -6.805   5.023  -0.361                         H
HETATM   51  H           1      -6.094   5.543  -1.982                         H
HETATM   52  H           1       4.411  -4.914  -2.517                         H
```

```
HETATM   53  H           1       4.862  -4.678  -0.784                          H
HETATM   54  H           1       3.623  -5.939  -1.254                          H
CONECT    1    2    3    4
CONECT    2    1    5    6   29
CONECT    3    1    7    8
CONECT    4    1    9   32
CONECT    5    2   10   11
CONECT    6    2   12   33   34
CONECT    7    3   13    9
CONECT    8    3   11   35   36
CONECT    9    4    7   14
CONECT   10    5   15   37   38
CONECT   11    5    8   39   40
CONECT   12    6   16   15   31
CONECT   13    7   17   41
CONECT   14    9   18   42
CONECT   15   10   12   19   30
CONECT   16   12   20   21   43
CONECT   17   13   18   44
CONECT   18   14   17   22
CONECT   19   15   23   45   46
CONECT   20   16   24   23   47
CONECT   21   16   25   26
CONECT   22   18   27
CONECT   23   19   20   25   48
CONECT   24   20   28
CONECT   25   21   23
CONECT   26   21
CONECT   27   22   49   50   51
CONECT   28   24   52   53   54
CONECT   29    2
CONECT   30   15
CONECT   31   12
CONECT   32    4
CONECT   33    6
CONECT   34    6
CONECT   35    8
CONECT   36    8
CONECT   37   10
CONECT   38   10
CONECT   39   11
CONECT   40   11
CONECT   41   13
CONECT   42   14
CONECT   43   16
CONECT   44   17
CONECT   45   19
CONECT   46   19
CONECT   47   20
CONECT   48   23
CONECT   49   27
CONECT   50   27
CONECT   51   27
CONECT   52   28
CONECT   53   28
CONECT   54   28
END
```

Coordinates of RD-1 in coo-file format for the correct configuration

The coordinates of RD-1 in coo-file format generated for the program COSMOS with the correct configuration is given below.

```
$COO006
CELL  1.0000  1.0000  1.0000  90.000  90.000  90.000  0.0000  0.0000  0.0000
NAME  ZTM_11_D
ATOMS 54
O25 8  4.93307  8.85633  0.63735 mbo 1 0 24 7 0 0 0 0 0 0 1
O24 8 16.13042  5.63958 -2.15140 mbo 1 0 26 8 0 0 0 0 0 0 1
O1 8 15.70592  5.31858  1.40838 mbo 1 0 28 27 0 0 0 0 0 0 1
O27 8 13.52503  4.64242  1.70257 mbo 1 1 27 0 0 0 0 0 0 0 1
N13 7  9.71428  7.70847  0.49333 mbo 1 6 32 15 9 0 0 0 0 0 1
N8 7 13.16829  8.87841  1.40679 mbo 1 0 17 16 10 0 0 0 0 0 1
C26_CH3 6  3.92393  9.72713  1.13377 mbo 1 0 51 50 49 1 0 0 0 0 1
C28_CH3 6 17.41463  5.22365 -2.59511 mbo 1 0 54 53 52 2 0 0 0 0 1
C12 6 10.82355  8.29122  1.08034 mbo 1 5 11 10 5 0 0 0 0 0 1
C23 6 12.24198  7.81557  0.96726 mbo 1 0 29 12 9 6 0 0 0 0 1
C11 6 10.42153  9.35635  1.83256 mbo 1 6 14 13 9 0 0 0 0 0 1
C22 6 12.61310  7.42668 -0.47754 mbo 1 0 34 33 18 10 0 0 0 0 1
C15 6  8.99641  9.45850  1.69504 mbo 1 7 19 15 11 0 0 0 0 0 1
C10 6 11.41377 10.17729  2.59183 mbo 1 0 36 35 17 11 0 0 0 0 1
C14 6  8.56828  8.40054  0.83874 mbo 1 7 20 13 5 0 0 0 0 0 1
C7 6 14.52596  8.34463  1.50670 mbo 1 0 38 37 21 6 0 0 0 0 1
C9 6 12.76103  9.42136  2.71574 mbo 1 0 40 39 14 6 0 0 0 0 1
C5 6 14.08066  6.98428 -0.66585 mbo 1 0 31 22 21 12 0 0 0 0 1
C16 6  8.00101 10.32674  2.19601 mbo 1 6 41 23 13 0 0 0 0 0 1
C19 6  7.21474  8.21974  0.50254 mbo 1 6 42 24 15 0 0 0 0 0 1
C6 6 15.06078  7.91191  0.11922 mbo 1 0 30 25 18 16 0 0 0 0 1
C4 6 14.34562  5.45590 -0.46013 mbo 1 0 43 27 26 18 0 0 0 0 1
C17 6  6.64143 10.15201  1.86258 mbo 1 6 44 24 19 0 0 0 0 0 1
C18 6  6.23591  9.09493  1.01146 mbo 1 3 23 20 1 0 0 0 0 0 1
C21 6 16.49054  7.30843  0.22733 mbo 1 0 46 45 28 21 0 0 0 0 1
C3 6 15.78158  5.12391 -0.86523 mbo 1 0 47 28 22 2 0 0 0 0 1
C20 6 14.44901  5.07582  1.01321 mbo 1 2 22 4 3 0 0 0 0 0 1
C2 6 16.52013  5.76040  0.32437 mbo 1 0 48 26 25 3 0 0 0 0 1
H23 1 12.35444  6.95114  1.62335 mbo 1 0 10 0 0 0 0 0 0 0 1
H6 1 15.15148  8.83647 -0.45406 mbo 1 0 21 0 0 0 0 0 0 0 1
H5 1 14.27983  7.16172 -1.72288 mbo 1 0 18 0 0 0 0 0 0 0 1
H13 1  9.76290  6.89016 -0.10250 mbo 1 0 5 0 0 0 0 0 0 0 1
H22B 1 11.94849  6.64305 -0.84180 mbo 1 0 12 0 0 0 0 0 0 0 1
H22A 1 12.42704  8.28915 -1.11947 mbo 1 0 12 0 0 0 0 0 0 0 1
H10A 1 11.57220 11.12865  2.08143 mbo 1 0 14 0 0 0 0 0 0 0 1
H10B 1 11.03421 10.42351  3.58462 mbo 1 0 14 0 0 0 0 0 0 0 1
H7A 1 15.18579  9.11796  1.90556 mbo 1 0 16 0 0 0 0 0 0 0 1
H7B 1 14.55318  7.52621  2.22632 mbo 1 0 16 0 0 0 0 0 0 0 1
H9B 1 12.66928  8.60676  3.43635 mbo 1 0 17 0 0 0 0 0 0 0 1
H9A 1 13.51861 10.10344  3.10614 mbo 1 0 17 0 0 0 0 0 0 0 1
H16 1  8.29309 11.13840  2.84809 mbo 1 0 19 0 0 0 0 0 0 0 1
H19 1  6.92672  7.40640 -0.14821 mbo 1 0 20 0 0 0 0 0 0 0 1
H4 1 13.62421  4.83566 -0.99153 mbo 1 0 22 0 0 0 0 0 0 0 1
H17 1  5.92720 10.85033  2.27912 mbo 1 0 23 0 0 0 0 0 0 0 1
H21B 1 17.06116  7.60325 -0.65404 mbo 1 0 25 0 0 0 0 0 0 0 1
H21A 1 17.02490  7.74364  1.07265 mbo 1 0 25 0 0 0 0 0 0 0 1
```

```
H3 1 15.92005  4.03946 -0.86823 mbo 1 0 26 0 0 0 0 0 0 0 1
H2 1 17.54076  5.38710  0.41886 mbo 1 0 28 0 0 0 0 0 0 0 1
H26C 1  3.87139  9.69909  2.22318 mbo 1 0 7 0 0 0 0 0 0 0 1
H26B 1  4.08633 10.75561  0.80787 mbo 1 0 7 0 0 0 0 0 0 0 1
H26A 1  2.95314  9.41086  0.75105 mbo 1 0 7 0 0 0 0 0 0 0 1
H28B 1 17.61296  5.63980 -3.58262 mbo 1 0 8 0 0 0 0 0 0 0 1
H28C 1 18.20378  5.56946 -1.92656 mbo 1 0 8 0 0 0 0 0 0 0 1
H28A 1 17.47527  4.13685 -2.67305 mbo 1 0 8 0 0 0 0 0 0 0 1
```

Data file for COSMOS MD simulation (.cod) for RD-1

The cosmos data file (.cod) containing the experimental data for RD-1 prepared for the MDOC run in the program COSMOS is shown below. The file contains at the beginning lines with remarks (REMARK), which contain information about the conditions and the origin of data, the used groups and their meaning. The experimental RDCs used as constraints follow (DD_TENSORS), showing the used grouping (GROUP_MEMBERS), and fixing of distances (FIXED_DISTANCES).

Beginning of the COSMOS data file

```
$COD003
REMARK NMR parameters for ZTM002751
REMARK Measurements of Pavleta and Emine - Conditions:

REMARK COD as simplified as possible (USt)
REMARK --------------------------------------------------------------------
REMARK Group 1: all atoms (not used)
REMARK Group 2: bond DD tensors - used as constraints (only one atom (H) has to be in group 2)
REMARK Group 4: Torsion angles for Mathematica snapshot
REMARK Group 5:
REMARK Group 6:
REMARK Group 7: fixed distances (for force field)
REMARK Group 8: Fix CH-bond lengths (all H atoms - not used)
REMARK --------------------------------------------------------------------
REMARK H26[ABC] and H28[ABC] are given the constraints of the CH3-protons

REMARK That creates in MDOC an rotating CH3-group
NAMES_OPT_FIT_TO EXACT
DATA_OPT_END
DD_TENSORS 21
H10B    C10           -7.17         -7.17         14.34         0      0      0(5)
H10A    C10           -11.37        -11.37        22.74         0      0      0(5)
H6      C6            -12.92        -12.92        25.84         0      0      0(1)
H21B    C21           -9.1          -9.1          18.20         0      0      0(0.3)
H21A    C21           1.99          1.99          -3.98         0      0      0(0.3)
H5      C5            -12.045       -12.045       24.09         0      0      0(1.3)
H22A    C22           -12.69        -12.69        25.38         0      0      0(0.3)
H22B    C22           3.77          3.77          -7.54         0      0      0(0.3)
H4      C4            6.92          6.92          -13.84        0      0      0(0.6)
H9A     C9            6.665         6.665         -13.33        0      0      0(0.3)
H9B     C9            -16.025       -16.025       32.05         0      0      0(1.5)
H23     C23           -13.365       -13.365       26.73         0      0      0(0.3)
H7B     C7            -13.195       -13.195       26.39         0      0      0(5)
H7A     C7            8.285         8.285         -16.57        0      0      0(5)
H26[ABC] C26_CH3      -0.715        -0.715        1.43          0      0      0(0.3)
H28[ABC] C28_CH3      -1.545        -1.545        3.09          0      0      0(0.4)
H3      C3            -2.475        -2.475        4.95          0      0      0(0.3)
H2      C2            12.825        12.825        -25.65        0      0      0(1.8)
H19     C19           2.62          2.62          -5.24         0      0      0(0.3)
H17     C17           9.71          9.71          -19.42        0      0      0(1)
H16     C16           3.05          3.05          -6.1          0      0      0(1)


FIXED_DISTANCES 3
C9 C23
```

```
C9 C7
C7 C23
GROUP_MEMBERS 24
H[23456791].*$        1 all atoms
C[234567891].*$              1
N[1].*$                      1
O[12].*$                     1
H1[679]               2 bond DD tensors (constraints)
H[34569].*$                  2
H2[12][AB]            2
H23                          2
H2                           2
H7B                          2
H7A                          2
H10B                         2
H10A                         2
H26[ABC]                     2
H28[ABC]                     2
C[2345679]                   4 Torsion angles for Mathematica snapshot
C1[01245]                    4
C2[0123]                     4
N13                          4
N8                           4
O1                           4
C[79]                        7 Fixed distances (for Force Field)
C23                          7
H.*$                         8 Fix CH-bonds (not used)
END
```

End of COSMOS data file

Data file for MSpin SVD fitting calculation for RD-1

The data file to be used with the program MSpin to perform the singular value decomposition (SVD) calculation as a comparison to the outcome from the MD simulation is given below. It contains the same experimental data, but the data file format is different to the COSMOS input data file. For easy readability of the data file the atom pair labels are given as a remark, marked with # symbol.

Beginning of the MSpin data file

```
rdc_data {
#H10B   C10
23      48      14.34   5
# H10A  C10
23      47      22.74   5
#H21A   C21
12      37      -3.98   0.3
#H21B   C21
12      38      18.2    0.3
#H5     C5
19      52      24.09   1.3
#H6     C6
16      53      25.84   1
#H22A   C22
25      50      25.38   0.3
#H22B   C22
25      49      -7.54   0.3
#H4     C4
15      40      -13.84  0.6
#H9B    C9
20      43      32.05   1.5
#H9A    C9
2044    -13.33  0.3
#H7B    C7
21      45      26.39   5
#H7A    C7
21      46      -16.57  5
#H23    C23
27      54      26.73   0.3
#H2     C2
9       35      -25.65  1.8
#H3     C3
11      36      4.95    0.3
#H19    C19
17      41      -5.24   0.3
#H17    C17
14      39      -19.42  1
#H16    C16
18      42      -6.1    1
}
```

End of MSpin data file

MDOC run Project file (.cos) for RD-1

The program COSMOS requires a project file, called cosmos options file (.cos), which contains all the parameters, which define the MD simulation. The project file allows starting the MD simulations in the background and/or on a cluster environment. The project file for RD-1 is given below.

Beginning of the project file

```
//----------------------------------------------------------------------------
// COSMOS Project File for Version: 6
//----------------------------------------------------------------------------
// File Names
//----------------------------------------------------------------------------
PSE_FILE cosmos.pse             //  Periodic System of Elements
FF_PARMFILE forces.par          //  File with non standard force field parameters. If the entry
is empty or forces.par: dafault parameters are used•
Q_PARMFILE q631gnbo.cha         //  Parameters for BPT-charge calculation
BPT_PARMFILE HC_CC-DD.pol       //  Parameters for BPT-property calculation
LOG_FILE COSMOSLog.txt          //  File for Log data output (default: COSMOSLog.txt) (backend)
OUTPUT_DIR C:\cdev\COSMOS\exec\OUTPUT\ //  Blank|Directory for output files (front end)
DATA_IMPORT_FILE                //  Blank|Read NMR data and constraints from this *.cod or
*.coo-file (backend)
//----------------------------------------------------------------------------
// Bond Search Parameters
//----------------------------------------------------------------------------
BondSearch 0                    //  0|1:Bond search (front end)
MultipleBondSearch 0            //  0|1:Multiple bond search (front end)
HydrogenBondSearch 0            //  0|1:Hydrogen bond search (front end)
BondLengthFactor 0.1            //  r(A-B)<rad(A)+rad(B)+BondLengthFactor ? bond:true
DoubleBondValence 1.39          //  Valence>DoubleBondValence ? double bond:true
TripleBondValence 2.39          //  Valence>TripleBondValence ? triple bond:true
AddDistForHydrogenBonds 1.6     //  r(A-B)<rad(A)+rad(B)+AdditionalDistanceForHydrogenBonds ? H
bridge:true
//----------------------------------------------------------------------------
// Calculation of Atomic Charges
//----------------------------------------------------------------------------
ChargeCalcMode 2                //  Mode = 0: No Calculation | 1: EN-Charge | 2: BPT-Charge
Q_CutoffRadius 30               //  Cutoff radius for the charge calculation
ChargeIncludeSet 0              //  0|n<25 Calculate charges only for this group n
ChargeExcludeSet 0              //  0|n<25 Exclude this group n from charge calculation
ChargedMolecule 0               //  0|1 Search for charged groups in peptides and proteins
pH_Value 7                      //  pH_Value for charged group search
//----------------------------------------------------------------------------
// Calculation of Atomic Properties using the Bond Polarization Theory
//----------------------------------------------------------------------------
BPT_CutoffRadius 30             //  a>0.0: Cutoff radius for BPT property calculations
PCalc_IncludeSet 2              //  0|n<25 calculate properties for this group - note: only one
atom has to be within this group
PCalc_ExcludeSet 0              //  0|n<25 exclude this group from property calculation - only
one atom has to be within this group
CS-References C-19:ppm/CFCl3  0.000  //  blank|Text with CS references: form El-Mn:name value
(C-13:ppm/TMS 0.0)
BPT_PolIncludeSet 0             //  0|n<25 use only this group for bond polarization
BPT_PolExcludeSet 0             //  0|n<25 exclude this group from bond polarization
```

```
TensorCalcMode 1               //  1: Calculate full tensor | 2: Oriented sample average | 3:
Isotropic mean values
SetTensorPoints 2              //  0|n<25 calculate local order tensors for this group
PropertyKeyNumbers 1           //  n>0 Numbers of selected data types for the property
calculation
//-------------------------------------------------------------------------------
// Parameters for the COSMOS-NMR Force Field
//-------------------------------------------------------------------------------
BondLengthOpt 1                //  0|1 Optimize bond length
BondAngleOpt 1                 //  0|1 Optimize bond angles
PiBondTorsionFactor 1          //  a>0 Factor to enhance the pi-torsion barrier (default 1.0)
DiederAngleOpt 1               //  0|1 Optimize torsion angles
VanDerWaalsOpt 1               //  0|1 Optimize Van der Waals energy
TruncVDW_Repuls 0              //  0|1 Truncate the VdW repulsion to 48 times min value
ElectrostaticOpt 1             //  0|1 Optimize electrostatic energy
BendAngleOpt 1                 //  0|1 Optimize bend angles
AllNBInteractions 1            //  0|1 include all interactions of fixed groups
NB_ElectrostaticsOnly 0        //  0|1 include only electrostatics of fixed groups (version 6)
ELS_ForceMemoryTimeC 100       //  NB_ElectrostaticsOnly?: Memory time (ps) for electrostatic
forces
FF_IncludeSet 0                //  0|n<25 run force field for this connected group only
FF_ExcludeSet 0                //  0|n<25 exclude this connected group from force field
FF_FixSet 0                    //  0|n<25 fix this group of atoms
FF_FixedBondsSet 1             //  0|n<25 fix all bond lengths for this group in MD
simulations
MD_FixedDistancesSet 7         //  0|n<25 fix distances as given in R-constraints for this
group in MD simulations(version 6)
FF_UnitCellSet 0               //  0|n<25 group of unit cell content
CutoffRadVDW 6                 //  Cutoff radius for Van der Waals interactions
CutoffRadELS 30                //  Cutoff radius for electrostatic interactions
HarmonicBondPotential 1        //  0|1: Use harmonic potentials for bond forces
PeriodicBoundaryCond 0         //  0|1: Periodic box
CrystBoundaryCond 0            //  0|1: Periodic crystal lattice
BPT-PeriodicCell/Box 0         //  0|1: BPT calculation for periodic box or cell
ShiftMolToCrystCell 0          //  0|1: Shift molecules to cell in MD calculations
//-------------------------------------------------------------------------------
// Geometry Optimization using the COSMOS-NMR Force Field
//-------------------------------------------------------------------------------
MaxCycles 1000                 //  n: Maximum number of cycles
PictureCycles 1                //  0|n: Refresh picture after n cycles (front end)
ControlCycles 10               //  0|n: Generate log output after n cycles (front end)
ChargeCycles 1                 //  0|n: Calculate BPT charges after n cycles
OptimizationMode 1             //  0: Gradient optimization | 1: conjugated gradient
optimization
StartSlope 1                   //  a>0 Optimization step width at start (default 1.0)
ForceEps 1e-06                 //  fabs(forces(cycle n)-forces(cycle n-1))<eps ?: terminate
optimization
//-------------------------------------------------------------------------------
// Snapshots in Molecular Dynamics Simulations using the COSMOS-NMR Force Field
//-------------------------------------------------------------------------------
SnapshotTypes COO TORSION DD_TENS //  TYPE TYPE
...TYPE=COO|DIST|ANGLES|TORSION|RAMA|INERTIA|CHARGES|CS_ISO|CS_TENS|DD_TENS|QC_TENS|RDC|JC|ENE
RGY
COO_SnapGroup 0                //  0|n<25 Group for coordinate (COO) snapshot
DIST_SnapGroupFrom 1           //  0|n<25 Distance (DIST) snapshots from this group n of atoms
DIST_SnapGroupTo 1             //  0|n<25 Distance (DIST) snapshots to this group n of atoms
DIST_SnapCutoff 4              //  a>0.0 Cutoff radius a for distance (DIST) snapshots
```

```
ANGLES_SnapGroup 0            //  0|n<25 Angles (ANGLES) snapshots from this group n of atoms
TORSIONS_SnapGroup 4          //  0|n<25 Torsion angles (TORSION) snapshots from this group n
of atoms
RAMA_SnapGroup 0              //  0|n<25 Ramachandran (RAMA) snapshots from this group n of
atoms
CHARGES_SnapGroup 0           //  0|n<25 Atomic charges (CHARGES) snapshots from this group n
of atoms
CS_SnapGroup 0                //  0|n<25 Chemical shift (CS_ISO) snapshots from this group n
of atoms
BPT_TensorGroup 0 2 0         //  0|n<25 BPT tensor (CS, DD, QC) snapshots from this group n
of atoms
RDC_SnapGroup 0               //  0|n<25 RDC-Coupling snapshots from this group n of atoms,
group 0:all calculated RDC
J_SnapGroup 6                 //  0|n<25 J-Coupling snapshots from this group n of atoms
//--------------------------------------------------------------------------------
// Property Calculation Controls in MD Simulations using the COSMOS-NMR Force Field
//--------------------------------------------------------------------------------
GroupFixedBondLength 1        //  0|n<25 Fix the bond length for this group n (set parameter
FF_FixedBondsSet for force field)
PropertyCycles 1              //  0|n: Calculate NMR properties after n cycles
EnablePropertyCalc 1          //  0|1: Enable BPT property calculation
Enable_NMR_Spec 0             //  0|1: Enable NMR spectra display (front end)
NMR_SpecCycles 10             //  n Cycles for NMR spectra refresh (front end)
TimeAverageProperty 1         //  0|1: Calculate time average of BPT properties
PropertyMemoryTimeConst 200   //  a>0.0 in pico sec: memory time constant for the property
average
//--------------------------------------------------------------------------------
// Temperature Controls in MD Simulations using the COSMOS-NMR Force Field
//--------------------------------------------------------------------------------
ThermostatingMode 1           //  0:No thermostating | 1:Keep temperature constant | 2:
Heating or cooling
RestartMode 1                 //  0:Restart at actual temperature | 1:Start with random
velocities
StartTemperature 300          //  a>0.0 Start temperature in Kelvin in the case of
RestartMode=1
TargetTemperature 300         //  a>0.0 Target temperature in Kelvin (heating or cooling)
T_CouplingTimeConst 0.02      //  a>0.0 Coupling time constant to the thermostat in pico sec
//--------------------------------------------------------------------------------
// Process Controls in MD Simulations using the COSMOS-NMR Force Field
//--------------------------------------------------------------------------------
DynaPicCycles 1000            //  0|n Cycles for the picture refresh (front end)
DynaControlCycles 200000      //  0|n Cycles for Log-window output or programmed stop
DataSnapshotCycles 40000      //  0|n Cycles for data snapshots
MaxTimeSteps 160000000        //  n>0 Maximum number of time steps
FemtoTimeStep 0.5             //  a>0.0 Verlet time step in femto sec.
DynChargeCycles 4             //  0|n Cycles for BPT atomic charge calculation
CoordSnapshotCycles 80000     //  0|n Cycles for coordinate snapshots
HeatingCoolingCycles 298      //  0<n<=MaxTimeSteps: Cycles for heating or cooling (only if
ThermostatingMode==2)
//--------------------------------------------------------------------------------
// MPI Controls in MD Simulations using the COSMOS-NMR Force Field
//--------------------------------------------------------------------------------
GlobalRemoveTransRotCyc 1000  //  n Cycles for global remove of translations or rotation (MPI
backend only)
GlobalThermostatingCyc 1000   //  n Cycles for global thermostating (MPI backend only)
GlobalExchangeChargesCyc 100  //  n Cycles for global BPT charge exchange (MPI backend only)
ProcessPartitionMethod 0      //  0:Process partition of molecules |1:Sequential partition
```

```
//---------------------------------------------------------------------------
// Spectra and Distributions Controls in MD Simulations using the COSMOS-NMR FF
//---------------------------------------------------------------------------
SaveNMR_Spectra 0               //  n Cycles to save NMR spectra (front end)
SaveDistributionFkt 0           //  n Cycles to save distribution functions (front end)
EnableCalcDistFkt 0             //  0|1: Enable the calculation of distribution functions
(front end)
EnableShowDistFkt 0             //  0|1: Enable the display of distribution functions (front
end)
EnableResetMotions 1            //  0|1: Enable the elimination of global motions
PutAllMoleculesToBox 0          //  1: All molecules are put to the periodic box |0: Mol. with
atoms outside the box are not treated periodically
//---------------------------------------------------------------------------
// Table Output
//---------------------------------------------------------------------------
AtNbInCoordTable 1              //  0|1: Numbering of atoms in table
SymbInCoordTable 1              //  0|1: Site name in table
PseNbInCoordTable 1             //  0|1: PSE number in table
CoordTypeInTable 0              //  0: Cartesian | 1: crystallographic coordinates (front end)
AtNbInChargeTable 1             //  0|1: Numbering in charge table
SymbInChargeTable 1             //  0|1: Site name in charge table
PseNbInChargeTable 1            //  0|1: PSE number in charge table
CSIsoTable 1                    //  0|1: Display isotropic property table
CSTensorTable 0                 //  0|1: Display tensorial property table
CSAnisoTable 1                  //  0|1: Display anisotropy parameters
SelectedTableType 0             //  0: All atoms | 1: Group | 2: Surrounding of atom in
property table
CentralAtInTable 1              //  0|n: Central atom n for surrounding for table type 2
GroupInTable 4                  //  0|n: Group n for table type 1
RadiusInTable 1e+03             //  r>0 Radius for atom surrounding for table type 2
GeoOptEnergyLog 1               //  0|1: Output of running geometry optimization to log-window
(front end) or log-file
DynamicsOutputLog 1             //  0|1: Output of running MD info to log-window (front end) or
log-file
MonteCarloOutputLog 0           //  0|1: Output of Monte-Carlo info to log-window (front end)
or log-file
TableOutputType CONSTR RCON JCON RDC ORDER //  TYPE TYPE...TYPE=CHAR|BPT-PROP|JC|TENS-
O|PISEMA|ORDER|DIP|CONSTR|RDC|RCON|JCON|PSEUDO-EN|ENERGY
SkipTimeMeanValue 1e+03         //  a>=0: Time in pico seconds that has to skipped from MD
start in mean value calculations (backend only)
//---------------------------------------------------------------------------
// Fit of Molecular Structures to Properties and NMR-Spectra
//---------------------------------------------------------------------------
ChemicalShiftWidth 1            //  a>0.0 Width parameter for the fit to property constraints
CS_WeightFactor 1               //  a>0.0 Weight factor for the BPT pseudo forces
//---------------------------------------------------------------------------
// Calculation of BPT Pseudo-Forces for NMR Structure Fit
//---------------------------------------------------------------------------
CalculateDerivatives 0          //  0|1: Calculate BPT property derivatives
FitToNMR_Properties 1           //  0|1: Fit To NMR properties
NMR_ExperimentType 0            //  0: Fit to isotropic val. | 1: Fit to principal tensor comp.
| 2: Fit to full symmetric tensor
NMR_FitOnlyForGroup 0           //  0|n<25: Group for fit to properties
//---------------------------------------------------------------------------
// BPT Pseudo-Forces for Orientational CS TensorFit
//---------------------------------------------------------------------------
NMR_FitExcludeGroup 0           //  0|n<25: Exclude group from fit to properties
```

```
CalcCS_OrientDeriv 0           //  0|1: Calculate orientational property derivatives
GroupOnlyCS_Orient 0           //  0|n<25: Group for fit to orientational properties
ExcludeGroupCS_Orient 0        //  0|n<25: Exclude group from fit to orientational properties
CS_OrientExpType 0             //  0: Fit oriented to zz-comp. | 1:Fit oriented to principal
tens. | 2: Fit oriented to full tensor
//--------------------------------------------------------------------------------
// BPT Pseudo-Forces for Orientational Dipolar Tensor Fit
//--------------------------------------------------------------------------------
CalcDD_OrientDeriv 1           //  0|1: Calculate dipolar oriented derivatives
GroupOnlyDD_Orient 0           //  0|n<25: Group n for DD orientational fit
ExcludeGroupDD_Orient 0        //  0|n<25: Exclude group n from DD orientational fit
//--------------------------------------------------------------------------------
// BPT Pseudo-Forces for Orientational Quadrupolar Tensor Fit
//--------------------------------------------------------------------------------
DD_OrientExpType 2             //  0: Fit DD oriented to zz-comp. | 1:Fit DD oriented to
principal tens. | 2: Fit DD oriented to full tensor
CalcQ_OrientDeriv 0            //  0|1: Calculate quadrupolar orientational derivatives
GroupOnlyQ_Orient 0            //  0|n<25: Group n for quadrupolar orientational fit
ExcludeGroupQ_Orient 0         //  0|n<25: Exclude group n from quadrupolar orientational fit
//--------------------------------------------------------------------------------
// Time Constants tau for the 1-exp(-t/tau) Rise of BPT Pseudo-Forces
//--------------------------------------------------------------------------------
Q_OrientExpType 0              //  0: Fit Q oriented to zz-comp. | 1:Fit Q oriented to
principal tens. | 2: Fit Q oriented to full tensor
PsForceReactionTime 200        //  tau>0 in pico sec: 1-exp(t/tau) rise time for orientational
pseudo forces (MD)
IsoPropertyReactTime 100       //  tau>0 in pico-sec: Time constant tau  for the 1-exp(t/tau)
rise of the isotropic pseudo forces (MD)
//--------------------------------------------------------------------------------
// Width and Weight Parameters for Distance Constraints
//--------------------------------------------------------------------------------
FitToR_Constrains 0            //  0|1: Fit to distance constraints
R_ConstrainsWidth 0.5          //  a>0 Width parameter for the distance constraints in
Angstoem
R_ConstrainsWeightFactor 0.03  //  a>0 Weight factor for the distance pseudo forces
R_ConstrainsIncGroup 0         //  0|n<25: Group for the distance constraints fit - both atom
have to be in this grup
R_ConstrainsExcGroup 0         //  0|n<25: Exclude this group from distance constraint fit -
only one atom has to be in this group
R_MemoryTimeConstant 200       //  0|a>0 Memory time constant for the NOE distance time
average
NOE_MeanValueMode 6            //  3|6: Mode of NOE average 3: (1/r^3) average, 6: (1/r^6)
average (default)
//--------------------------------------------------------------------------------
// Width and Weight Parameters for Orientational Pseudo-Forces
//--------------------------------------------------------------------------------
CS_TensorWidth 0               //  a>0 Width parameter for the oriented CS constraints fit
CS_TensorWeight 0              //  a>0 Weight factor for the oriented CS pseudo forces
DD_TensorWidth 0.5             //  a>0 Width parameter for the oriented DD constraints fit
DD_TensorWeight 0.00055         //  a>0 Weight factor for the oriented DD pseudo forces
Q_TensorWidth 0                //  a>0 Width parameter for the oriented QC constraints fit
Q_TensorWeight 0               //  a>0 Weight factor for the oriented QC pseudo forces
//--------------------------------------------------------------------------------
// Calculation of Distribution Functions
//--------------------------------------------------------------------------------
DistanceDistrDataPoints 600    //  n: Number of data points in distance distribution graphics
CalcDistDitribution 0          //  0:1 Calculate distance distribution (front end V6)
```

```
DistanceDistrSearchRad 6       //  a>0 Search radius for distance distribution
DistanceGroup 0                //  0|n<25 Group for distance distribution
DistanceGroupTo 0              //  0|n<25 Second group for distance distribution
CalcBondAnglesDist 0           //  0:1 Calculate bond angle distribution graphics (front end
V6)
BondAngleGroup 0               //  0|n<25 Group for bond angle distribution
BondAngleMin 0                 //  a: Minimum in bond angle graphics
BondAngleMax 180               //  a: Maximum in bond angle graphics
CalcTorsionAnglesDist 0        //  0:1 Calculate torsion angle distribution graphics (front
end V6)
TorsionAngleGroup 0            //  0|n<25 Group for torsion angle distribution
TorsionAngleMin -180           //  a: Minimum in torsion angle graphics
TorsionAngleMax 180            //  a: Maximum in torsion angle graphics
BondAngleDistType 0            //  0: General angle distribution | 1: Bond angle distribution
//--------------------------------------------------------------------------------
// Display of Chemical Shift Spectra
//--------------------------------------------------------------------------------
Display_CS_Spectrum 0          //  0|1: Display CS spectrum (front end)
CS_SpectrumMode 0              //  0: Isotropic chemical shifts | 1: zz-component of CS tensor
| 2: powder pattern
CS_NumberDataPoints 1024       //  n: Number of data points in CS spectrum graphics
CS_SpecXMin 0                  //  a: Minimum CS value in spectrum graphics in ppm
CS_SpecXMax 100                //  a: Maximum CS value in spectrum graphics in ppm
CS_UseSpecMaxMin 0             //  0: Automatic minimum/maximum search | 1: Use
maximum/minimum values
//--------------------------------------------------------------------------------
// Display of Quadrupolar Spectra
//--------------------------------------------------------------------------------
Display_QC_Spectrum 0          //  0|1: Display quadrupolar spectrum (front end V6)
QC_SpectrumMode 1              //  1: zz-component of QC tensor | 2: powder pattern
QC_NumberDataPoints 1024       //  n: Number of data points in QC spectrum graphics
QC_SpecXMin -50                //  a: Minimum QC value in spectrum graphics in kHz/Hz
QC_SpecXMax 50                 //  a: Maximum QC value in spectrum graphics in kHz/Hz
QC_UseSpecMaxMin 0             //  0: Automatic minimum/maximum search | 1: Use
maximum/minimum values
//--------------------------------------------------------------------------------
// Display of Dipolar Spectra
//--------------------------------------------------------------------------------
Display_DD_Spectrum 0          //  0|1: Display dipolar spectrum (front end V6)
DD_SpectrumMode 1              //  1: zz-component of DD tensor | 2: powder pattern
DD_NumberDataPoints 1024       //  n: Number of data points in DD spectrum graphics
DD_SpecXMin -50                //  a: Minimum DD value in spectrum graphics in kHz/Hz
DD_SpecXMax 50                 //  a: Maximum DD value in spectrum graphics in kHz/Hz
DD_UseSpecMaxMin 0             //  0: Automatic minimum/maximum search | 1: Use
maximum/minimum values
//--------------------------------------------------------------------------------
// Width and Weight Factors of the RDC Distance Fit
//--------------------------------------------------------------------------------
FitToRDC_Distances 0           //  0|1: Fit to RDC distances
RDC_DistanceWidth 1            //  a>0 Width parameter for the residual dipolar distance
constraints in Angstoem
RDC_DistWeightFactor 0.01      //  a>0 Weight factor for the RD distance pseudo forces
RDC_DistanceIncGroup 5         //  0|n<25: Calculate RDC forces for group of constrained atoms
RDC_DistanceExcGroup 0         //  0|n<25: Exclude group of atoms from RDC force calculation
//--------------------------------------------------------------------------------
// Width and Weight Factors of the Orientational RDC Fit
//--------------------------------------------------------------------------------
```

```
CalcRDC_OrientDeriv 0          //  0|1: Calculate RDC orientational derivatives
RDC_OrientWidth 0.5            //  a>0 Width parameter for orientational residual dipolar
constraints in Angstroem
RDC_OrientWeightFactor 0.001   //  a>0 Weight factor for the orientational RDC pseudo forces
RDC_OrientIncGroup 0           //  0|n<25: Calculate RDC orientational forces for 0:all or
group n of constrained atoms
RDC_OrientExcGroup 0           //  0|n<25: Exclude group of atoms from orientational RDC force
calculation
RDCForceReactionTime 200       //  tau>0 in pico sec: 1-exp(t/tau) rise time for RDC pseudo
forces (MD)
//--------------------------------------------------------------------------------
// RDC Calculation
//--------------------------------------------------------------------------------
Calculate_RDC 0                //  0|1: Calculate RDC for constrained atoms or group
RDC_Group 2                    //  0:Calculate RDC for all constrained atoms | n<25: for this
group n of atoms
RDC_MemoryTimeConstant 200     //  tau_mem>0 Memory time constant for the RDC time average
//--------------------------------------------------------------------------------
// Oriented Sample
//--------------------------------------------------------------------------------
OrientedTensCalcMode 1         //  0:None |1: Full tensor |2: Mean value for fast rotation
around director |3: Full tensor + rot. distribution
DirectorTiltAngle 0            //  0<= a <=180 Director tilt angle
SampleOrderParameter 0.004     //  0<= a <=1.0 Order parameter of oriented sample
DD_InteractionCalcMode 0       //  0|1 0: Calculate DD splittings (deafult) | 1: Calculate
Dipolar coupling constants
//--------------------------------------------------------------------------------
// Global Width Settings
//--------------------------------------------------------------------------------
UseErrorsAsWidth 1             //  0|1 Use the experimental errors as width for DD, RDC, R and
J constraints in MD simulations
//--------------------------------------------------------------------------------
// Import and Export Options
//--------------------------------------------------------------------------------
GaussianChargesImpMode 2       //  1:Q_POP_MUL | 2:Q_POP_NBO | 3:Q_POP_ESP
GaussianTensorImpMode 2        //  1:SCF GIAO Magnetic shielding tensors | 2:MP2 GIAO Magnetic
shielding tensors
GaussianCalcProc1 #t b3lyp/6-311G(d,p) pop(MK,NBO,dipole) #IOp(6/41=10) #IOp(6/42=10)
SCF=Tight
GaussianCalcProc2 #t mp2/6-311+G(d) nmr pop=none scf=tight
GaussianCalcProc3 #t b3lyp/6-311+G(2d,p) NMR Geom=AllCheck Guess=Read Test
TurbomoleCalcProc1 GEOMY :: b-p/SVP
TurbomoleCalcProc2 GEOMY :: ri-mp2/TZVPP
TurbomoleCalcProc3 GEOMY :: mp2/TZVP
//--------------------------------------------------------------------------------
// Parameters for 3J Couplings
//--------------------------------------------------------------------------------
SelectedJ_CalcParameter 3 4    //  n1 n2 ... ni 0<n<14 Selected Karplus parameter sets for the
3J coupling calculation
Calculate_J_Couplings 0        //  0|1 Calculate 3J couplings
Fit_ToJ_Couplings 0            //  0|1 Switch the fit to 3J couplings on
J_CouplingWidth 2              //  a>0 Width for the fit the 3J couplings in Hz
J_CouplingWeightFactor 0.4     //  a>0 Weight factor for the fit to 3J couplings
J_CouplingsCalcForGroup 4      //  0|n<25 Calculate 3J couplings for this group
J_CouplingsExcludeGroup 0      //  0|n<25 Exclude this group from the 3J calculation
JC_MemoryTimeConstant 200      //  0|a>0 Memory time constant for the 3J time average
```

End of the project file

MSpin and COSMOS results for all configurations of RD-1

Table S2: Experimental *vs.* calculated ${}^1D_{CH}$ values for all configurations of RD-1 obtained with MSpin. The 32 different configurations are listed using the numbering of Table 2. Number of outliers is provided in the last row.

	Exp	Err.	1	2	3	4	5	6	7	8	9	10	11	12	13	14	15	16	17	18
$C_{10}H_{10B}$	14.3	5.0	6.7	5.8	6.3	10.7	12.4	4.4	-1.9	-4.4	-0.2	-4.0	10.4	8.1	-6.1	-11.9	10.4	11.1	-3.5	6.9
$C_{10}H_{10A}$	22.7	5.0	13.9	24.2	24.4	27.3	26.5	16.4	9.8	8.8	-11.2	-7.0	23.7	22.1	-10.0	-9.1	29.1	28.9	-9.4	4.5
$C_{21}H_{21A}$	-4.0	0.3	-3.9	-1.1	-0.9	13.6	14.4	-3.8	-8.1	-9.9	-11.3	-15.3	-3.8	-3.2	6.3	2.4	10.5	10.6	-5.6	7.8
$C_{21}H_{21B}$	18.2	0.3	10.6	14.3	16.2	8.1	6.5	7.9	12.1	13.6	25.2	28.1	18.8	13.1	7.6	9.9	8.3	7.5	20.3	7.4
C_5H_5	24.1	1.3	13.5	11.1	13.8	28.1	28.1	10.7	19.4	19.9	15.3	13.4	27.2	22.3	11.2	9.1	20.0	19.2	-1.1	8.9
C_6H_6	25.8	1.0	26.7	7.7	9.0	22.4	21.7	24.9	18.3	19.8	17.2	17.6	25.7	24.1	18.3	23.7	22.6	22.2	7.2	3.5
$C_{22}H_{22A}$	25.4	0.3	-6.4	2.2	1.9	25.6	25.3	-7.6	32.8	33.9	15.7	15.0	24.3	24.2	13.6	14.4	7.9	7.2	8.7	8.5
$C_{22}H_{22B}$	-7.5	0.3	22.4	10.9	13.0	-5.7	-5.7	19.1	-18.1	-18.9	-6.3	-9.1	-11.2	-11.0	-3.0	-5.4	2.6	2.0	5.2	6.9
C_4H_4	-13.8	0.6	-3.5	3.9	3.8	-20.4	-20.0	-3.8	-9.8	-10.9	-5.3	-7.2	-13.9	-13.8	-12.9	-21.7	-6.2	-6.1	2.8	9.2
C_9H_{9B}	32.1	1.5	19.9	24.0	26.2	29.7	29.6	21.0	22.6	20.0	-3.3	-4.8	30.0	27.3	-0.8	-1.4	32.8	32.9	9.3	8.9
C_9H_{9A}	-13.3	0.3	-5.5	0.5	0.6	-13.0	-12.7	-6.8	-18.7	-19.0	7.1	6.5	-14.4	-14.4	0.3	1.6	-19.7	-19.5	1.4	10.6
C_7H_{7B}	26.4	5.0	26.9	4.2	3.5	26.1	25.8	24.8	29.3	26.3	11.4	9.4	29.3	27.5	16.1	18.9	26.7	27.3	8.4	8.6
C_7H_{7A}	-16.6	5.0	-2.9	9.1	10.7	-6.1	-6.1	-2.8	-17.7	-17.2	-4.9	-6.7	-11.3	-11.0	-1.9	-2.9	-2.6	-2.9	4.0	7.0
$C_{23}H_{23}$	26.7	0.3	19.6	8.8	10.7	26.2	25.9	16.3	32.4	31.4	11.8	12.1	26.2	25.6	5.0	9.5	27.9	27.4	5.6	6.4
C_2H_2	-25.7	1.8	-23.8	-28.4	-28.9	-11.5	-10.2	-24.2	-18.5	-15.4	-8.3	-13.7	-26.0	-23.3	-12.2	-24.4	-26.6	-26.2	-18.4	-17.2
C_3H_3	5.0	0.3	9.2	19.1	5.8	6.2	7.5	19.2	4.8	-3.5	18.0	-7.7	4.2	26.0	25.7	-2.9	5.8	7.9	-5.1	14.7
$C_{19}H_{19}$	-5.2	0.3	0.8	2.2	0.5	0.7	1.1	1.0	-2.5	-2.5	-2.3	-0.6	-5.4	-3.4	-7.5	-6.0	-7.1	-7.5	7.3	5.9
$C_{17}H_{17}$	-19.4	1.0	-19.2	-17.4	-22.0	-11.5	-13.0	-17.0	-17.4	-12.5	-8.7	-10.4	-16.6	-12.8	-6.6	-15.6	-9.2	-10.0	-19.9	-24.1
$C_{16}H_{16}$	-6.1	1.0	0.8	2.2	0.5	0.6	1.1	1.0	-2.5	-2.5	-2.2	-0.5	-5.4	-3.5	-7.3	-5.8	-7.2	-7.6	7.5	6.0
outliers			15	18	18	15	15	15	16	17	19	19	10	16	19	17	15	15	18	19

	Exp	Err.	19	20	21	22	23	24	25	26	27	28	29	30	31	32
$C_{10}H_{10B}$	14.3	5.0	6.0	3.5	0.1	-1.4	6.3	1.5	14.0	9.1	-2.9	-2.8	15.4	16.5	-14.1	-16.0
$C_{10}H_{10A}$	22.7	5.0	8.8	18.8	19.7	-13.2	28.1	29.1	-4.5	0.6	22.2	20.9	-3.4	0.5	9.6	12.8
$C_{21}H_{21A}$	-4.0	0.3	6.1	15.6	18.0	-2.6	6.5	8.8	0.3	4.7	-2.5	0.3	18.2	17.8	-4.8	-4.5
$C_{21}H_{21B}$	18.2	0.3	8.8	8.6	8.3	18.3	12.4	14.7	16.3	13.9	16.1	12.0	-4.2	-4.7	6.0	9.3
C_5H_5	24.1	1.3	10.3	23.1	23.4	0.6	16.9	17.1	2.5	-2.7	18.8	15.5	14.9	14.5	-1.4	1.7
C_6H_6	25.8	1.0	2.9	20.2	21.1	6.4	19.3	24.4	22.6	13.4	25.7	22.4	18.7	20.0	11.1	11.4
$C_{22}H_{22A}$	25.4	0.3	7.5	22.1	22.9	10.9	27.7	27.9	12.8	-3.0	24.2	22.9	15.3	14.7	-3.2	-2.3
$C_{22}H_{22B}$	-7.5	0.3	7.5	0.3	1.3	4.1	-4.4	-3.9	-15.2	1.5	-2.0	-1.8	-8.1	-10.6	3.0	4.7
C_4H_4	-13.8	0.6	8.6	-6.6	-3.3	4.6	1.9	1.7	-8.8	4.3	-4.0	-2.0	-5.1	-7.9	-2.9	-1.7
C_9H_{9B}	32.1	1.5	8.3	-3.1	-1.1	11.6	-4.5	-3.1	14.9	9.7	-2.2	-1.1	8.3	6.7	16.0	15.1
C_9H_{9A}	-13.3	0.3	11.8	8.8	6.0	0.1	7.5	2.5	8.2	10.4	9.5	8.1	11.8	13.6	-1.4	-1.5
C_7H_{7B}	26.4	5.0	7.7	21.6	22.2	10.7	23.7	23.1	14.4	9.4	27.8	25.3	17.2	17.4	-1.7	-1.4
C_7H_{7A}	-16.6	5.0	7.4	0.1	0.8	2.0	-3.6	-3.0	7.5	8.2	-3.5	-2.8	1.5	2.7	7.4	8.2
$C_{23}H_{23}$	26.7	0.3	6.7	22.6	23.0	4.0	22.2	22.0	2.5	5.2	26.4	23.8	7.3	7.3	22.5	20.3
C_2H_2	-25.7	1.8	-19.5	-10.7	-8.7	-10.7	-15.0	-14.0	-16.1	-20.6	-17.2	-14.0	-16.8	-20.0	-1.7	-0.2
C_3H_3	5.0	0.3	0.4	-4.6	5.6	16.6	-9.2	-3.4	12.1	-3.1	-3.7	24.8	13.0	-1.2	-0.8	-6.1
$C_{19}H_{19}$	-5.2	0.3	6.2	9.1	7.9	6.5	-5.3	-5.6	11.9	4.3	2.2	0.2	7.0	6.4	3.0	1.4
$C_{17}H_{17}$	-19.4	1.0	-27.5	-9.7	-10.2	-16.0	0.3	3.3	-14.0	-21.1	-19.6	-17.9	-12.5	-12.9	-17.6	-12.3
$C_{16}H_{16}$	-6.1	1.0	6.3	9.1	7.8	6.6	-5.3	-5.6	11.9	4.4	2.2	0.2	7.0	6.4	3.1	1.5
outliers			19	16	16	18	16	17	18	19	15	17	18	18	19	19

Table S3: Experimental *vs.* calculated $^{1}D_{CH}$ values for all configurations of RD-1 obtained with COSMOS. The 32 different configurations are listed using the numbering of Table 2. Number of outliers is provided in the last row.

	Exp	Err.	1	2	3	4	5	6	7	8	9	10	11	12	13	14	15	16	17	18
$C_{23}H_{23}$	26.7	0.3	23.5	23.7	24.2	26.6	26.7	24.1	26.3	26.4	25.5	25.4	26.6	26.6	23.0	22.9	24.3	23.8	22.6	21.2
C_6H_6	25.8	1.0	25.2	22.8	23.3	25.1	25.0	25.0	22.8	23.4	24.4	24.6	25.3	25.4	24.9	25.0	23.3	22.8	23.4	22.4
C_5H_5	24.1	1.3	22.9	22.1	22.6	24.0	24.1	21.9	22.3	22.7	22.1	20.4	23.4	22.8	23.2	23.0	22.6	22.1	20.7	21.6
$C_{22}H_{22B}$	-7.5	0.3	-7.0	-5.5	-5.2	-7.3	-7.2	-7.2	-8.0	-7.8	-7.7	-7.8	-7.5	-7.5	-7.5	-7.6	-5.4	-5.9	-6.9	-5.3
$C_{22}H_{22A}$	25.4	0.3	24.5	23.9	23.9	25.3	25.3	24.4	24.5	24.7	23.8	23.1	25.1	25.2	24.9	24.8	24.0	23.8	24.6	24.4
$C_{10}H_{10A}$	22.7	5.0	19.1	20.4	20.6	21.2	21.1	19.3	20.1	20.0	17.1	16.8	21.9	22.0	16.9	16.6	20.7	20.5	18.1	16.1
$C_{10}H_{10B}$	14.3	5.0	9.3	11.4	11.6	11.5	11.4	9.5	9.9	9.5	9.3	8.8	12.7	12.6	8.5	7.5	11.7	11.5	9.4	9.0
C_7H_{7A}	-16.6	5.0	-14.1	-6.3	-5.8	-12.4	-12.1	-14.2	-16.1	-15.6	-13.6	-13.8	-13.1	-13.1	-12.1	-12.5	-6.6	-7.4	-12.9	-6.6
C_7H_{7B}	26.4	5.0	25.9	22.3	21.9	26.8	26.9	25.7	24.3	24.8	23.6	22.7	26.8	27.0	25.1	24.8	22.1	22.7	24.9	23.4
C_9H_{9B}	32.1	1.5	28.3	30.2	30.5	31.6	31.6	28.2	30.6	30.8	29.5	29.5	31.6	31.6	28.4	28.2	30.6	30.3	30.5	31.1
C_9H_{9A}	-13.3	0.3	-13.1	-12.4	-12.4	-13.0	-12.9	-13.1	-13.4	-13.3	-12.3	-12.3	-13.1	-13.1	-13.0	-13.0	-12.3	-12.4	-12.1	-12.1
$C_{16}H_{16}$	-6.1	1.0	-5.9	-5.7	-5.7	-5.8	-5.8	-5.9	-6.0	-6.0	-5.9	-5.9	-5.9	-5.9	-6.0	-6.0	-5.7	-5.7	-5.8	-5.7
$C_{19}H_{19}$	-5.2	0.3	-5.2	-5.0	-5.0	-5.1	-5.1	-5.2	-5.3	-5.3	-5.2	-5.2	-5.1	-5.1	-5.2	-5.2	-5.0	-5.1	-5.0	-5.0
C_4H_4	-13.8	0.6	-13.6	-11.9	-12.6	-13.7	-13.5	-13.4	-13.8	-13.6	-13.6	-13.6	-13.5	-13.4	-13.3	-13.7	-12.6	-12.1	-13.4	-10.1
$C_{17}H_{17}$	-19.4	1.0	-19.0	-19.2	-19.3	-19.4	-19.5	-19.0	-19.0	-19.0	-19.1	-19.1	-19.3	-19.3	-18.8	-18.9	-19.3	-19.2	-19.3	-19.5
$C_{21}H_{21B}$	18.2	0.3	18.0	18.0	18.1	17.9	17.9	17.6	17.8	17.9	18.1	18.2	18.1	17.8	17.9	17.8	18.1	18.0	18.1	18.2
$C_{21}H_{21A}$	-4.0	0.3	-4.2	-3.6	-3.6	-3.4	-3.4	-4.2	-4.8	-4.5	-4.3	-4.2	-4.2	-4.2	-3.5	-3.7	-3.7	-3.7	-3.9	-3.5
C_3H_3	5.0	0.3	5.0	6.0	4.9	4.7	5.3	6.4	4.2	5.7	6.0	3.4	5.2	6.6	6.3	4.0	5.0	5.9	4.6	5.9
C_2H_2	-25.7	1.8	-24.5	-25.1	-25.8	-24.1	-23.6	-24.1	-16.7	-13.7	-14.7	-20.2	-25.7	-25.4	-23.4	-24.3	-25.7	-25.0	-23.9	-24.5
CH_{3-26}	1.4	0.3	1.3	1.3	1.3	1.3	1.3	1.3	1.3	1.3	1.3	1.3	1.3	1.3	1.3	1.3	1.3	1.3	1.3	1.3
CH_{2-28}	3.0	0.4	3.0	2.9	2.9	3.0	2.9	3.0	3.0	3.0	3.0	3.0	3.0	3.0	2.9	2.9	2.9	2.9	3.0	2.9
**outliers**			4	10	9	2	5	6	8	8	9	9	0	3	9	8	8	10	8	12

	Exp	Err.	19	20	21	22	23	24	25	26	27	28	29	30	31	32
$C_{23}H_{23}$	26.7	0.3	21.2	26.4	26.4	22.0	25.9	26.2	23.5	25.0	26.3	26.3	25.2	25.0	25.2	25.7
C_6H_6	25.8	1.0	22.8	25.0	25.0	23.2	23.1	24.0	24.5	24.7	25.2	25.1	24.7	24.9	22.4	22.5
C_5H_5	24.1	1.3	22.0	23.8	23.9	19.7	22.3	22.5	20.9	12.7	21.8	21.0	23.7	23.3	18.6	19.7
$C_{22}H_{22B}$	-7.5	0.3	-5.4	-6.9	-6.8	-7.0	-7.9	-7.6	-7.4	-7.8	-7.3	-7.4	-6.9	-7.1	-7.2	-7.2
$C_{22}H_{22A}$	25.4	0.3	24.4	25.3	25.3	24.5	24.5	24.7	24.4	23.7	24.5	24.4	25.3	25.2	18.1	20.5
$C_{10}H_{10A}$	22.7	5.0	17.2	21.4	21.2	17.5	20.7	20.2	18.5	18.7	20.8	20.7	18.4	18.4	18.9	19.5
$C_{10}H_{10B}$	14.3	5.0	9.5	12.3	11.9	9.3	10.3	9.1	9.9	10.0	11.2	11.0	10.4	10.3	9.3	9.7
C_7H_{7A}	-16.6	5.0	-6.4	-10.9	-10.8	-12.6	-14.8	-14.2	-9.6	-10.9	-13.6	-13.9	-10.9	-10.5	-11.1	-10.8
C_7H_{7B}	26.4	5.0	23.1	25.8	25.9	24.8	23.4	23.6	26.2	26.4	24.5	23.9	25.7	25.9	18.1	20.5
C_9H_{9B}	32.1	1.5	31.0	28.7	29.2	30.4	20.7	24.8	31.3	31.1	29.2	29.4	30.8	30.9	29.9	30.3
C_9H_{9A}	-13.3	0.3	-12.1	-12.0	-12.0	-12.2	-13.1	-13.0	-11.5	-11.7	-12.2	-12.4	-11.3	-11.6	-12.9	-12.6
$C_{16}H_{16}$	-6.1	1.0	-5.7	-5.7	-5.7	-5.8	-5.9	-5.9	-5.8	-5.9	-5.8	-5.8	-5.7	-5.7	-5.9	-5.8
$C_{19}H_{19}$	-5.2	0.3	-5.0	-5.0	-5.0	-5.1	-5.2	-5.2	-5.0	-5.1	-5.1	-5.0	-5.0	-5.0	-5.1	-5.1
C_4H_4	-13.8	0.6	-11.7	-13.5	-13.3	-12.9	-13.6	-13.5	-13.4	-13.5	-13.3	-13.0	-13.3	-13.4	-13.1	-12.5
$C_{17}H_{17}$	-19.4	1.0	-19.5	-19.3	-19.3	-19.3	-18.7	-18.8	-19.4	-19.3	-19.3	-19.3	-19.5	-19.4	-19.1	-19.2
$C_{21}H_{21B}$	18.2	0.3	18.2	18.0	18.0	18.0	17.9	18.0	18.2	18.2	18.0	17.8	18.1	18.2	18.0	18.0
$C_{21}H_{21A}$	-4.0	0.3	-3.6	-3.2	-3.2	-3.9	-4.5	-4.2	-4.0	-4.0	-4.0	-3.9	-3.5	-3.4	-4.0	-3.8
C_3H_3	5.0	0.3	4.6	4.8	5.4	6.2	3.9	5.8	5.7	4.2	5.0	6.4	5.5	4.5	4.2	5.6
C_2H_2	-25.7	1.8	-25.1	-24.2	-23.8	-23.1	-17.1	-12.9	-20.8	-22.9	-25.2	-24.6	-23.9	-24.5	-21.5	-21.4
CH_{3-26}	1.4	0.3	1.3	1.3	1.3	1.3	1.3	1.3	1.3	1.3	1.3	1.3	1.3	1.3	1.3	1.3
CH_{2-28}	3.0	0.4	2.9	3.0	2.9	3.0	2.9	2.9	2.9	3.0	3.0	3.0	2.9	2.9	3.0	2.9
**outliers**			11	6	8	11	9	9	8	8	5	8	7	6	13	12

Comparison of COSMOS *vs.* MSpin outliers

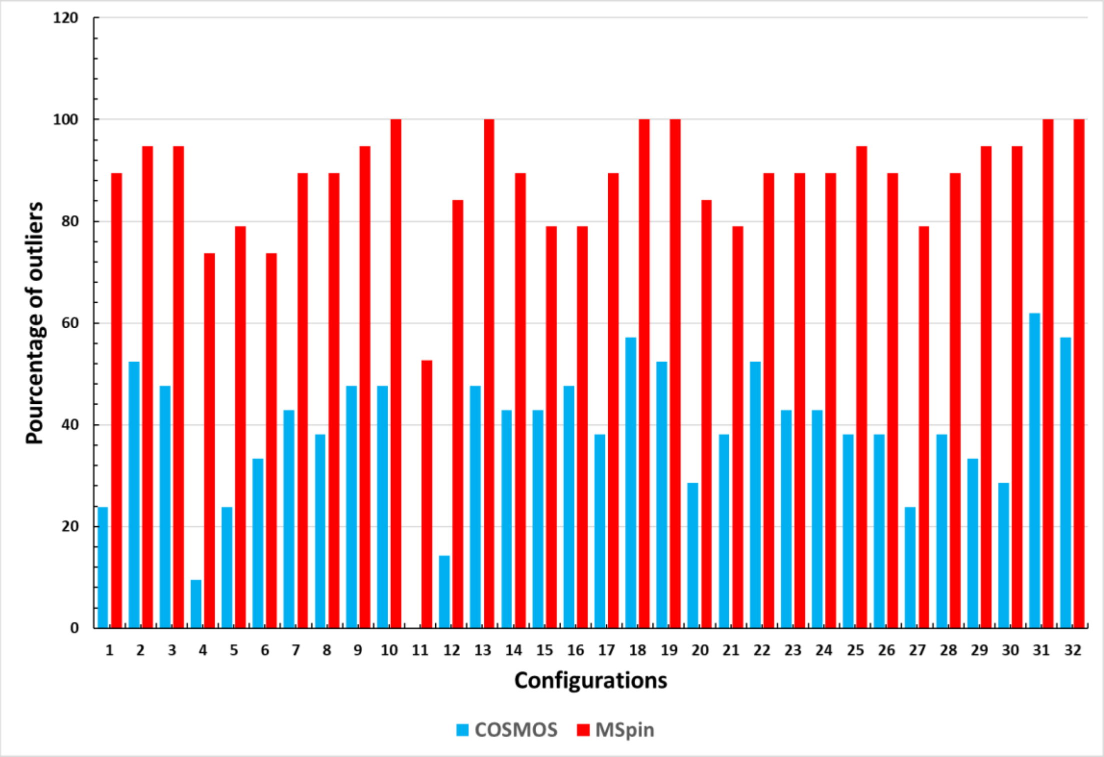

Figure S8: *Comparison of COSMOS (blue) vs. MSpin (Red) outliers in percentage*

On the horizontal axis, the 32 different configurations are listed using the numbering of Table 2. The configuration 11 has 0% of outliers with COSMOS and more than 50% with MSpin.

X-Ray: Crystallisation process

We took several times a point of spatula of substance (the equivalent of 1mg), placed it in a vial, and added 200 µl of various solvents. The vials were shaken until a clear solution was obtained (substance completely dissolved). If there were particles in suspensions, the suspension has been warmed up for a complete dissolution of the substance. The vials were then closed with an aluminum film with two small holes for slow evaporation.
The samples were then left at room temperature for several days for complete evaporation of the solvent. Crystals of the reserpine derivative RD-1 were obtained from tetrahydrofurane solution by slow evaporation of the solvent at room temperature.

Crystallographic data (excluding structure factors) have been deposited with the Cambridge Crystallographic Data Centre as supplementary publication number CCDC 1997972. Copies of the data can be obtained free of charge on application to CCDC, 12 Union Road, Cambridge CB2 1EZ, UK. Email: deposit@ccdc.cam.ac.uk. This material is available free of charge via the internet at http://pubs.acs.org.

Table S4. Crystal data and structure refinement for compound RD-1.

Identification code	ESA01a [#4829] [ZTM002751-NX-2] [THF]	
Empirical formula	$C_{22} H_{26} N_2 O_4$	
Formula weight	382.45	
Temperature	100(2) K	
Wavelength	1.54178 Å	
Crystal system	monoclinic	
Space group	$P2_1$	
Unit cell dimensions	*a* = 9.594(3) Å	α = 90°
	b = 7.583(3) Å	β = 96.958(15)°
	c = 13.165(4) Å	γ = 90°
Volume	950.7(6) Å^3	
Z	2	
Density (calculated)	1.336 g/cm^3	
Absorption coefficient	0.748 mm^{-1}	
F(000)	408	
Crystal size	0.21 x 0.12 x 0.03 mm^3	
Theta range for data collection	3.38 to 68.22°	
Index ranges	-11<=h<=11, -9<=k<=9, -15<=l<=15	
Reflections collected	20142	
Independent reflections	3478 [R(int) = 0.0358]	
Completeness to theta = 68.22°	100.0 %	
Absorption correction	semi-empirical from equivalents	
Max. and min. transmission	0.7530 and 0.6330	
Refinement method	full-matrix least-squares on F^2	

Data / restraints / parameters	3478 / 1 / 255
Goodness-of-fit on F^2	1.040
Final R indices [I>2sigma(I)]	R_1 = 0.0275, wR_2 = 0.0703
R indices (all data)	R_1 = 0.0284, wR_2 = 0.0713
Absolute structure parameter	0.02(13)
Largest diff. peak and hole	0.13 and -0.21 e·Å^{-3}
